# Combining potential and realized distribution modeling of telemetry data for a bycatch risk assessment

**DOI:** 10.1002/ece3.11541

**Published:** 2024-06-25

**Authors:** Bethany H. Frantz, Maritza Sepúlveda, Marisol García‐Reyes, Rodrigo Vega, Daniel M. Palacios, Luis Bedriñana‐Romano, Luis A. Hückstädt, Macarena Santos‐Carvallo, Jerry D. Davis, Ellen Hines

**Affiliations:** ^1^ School of the Environment San Francisco State University San Francisco California USA; ^2^ Centro de Investigación y Gestión de Recursos Naturales (CIGREN), Instituto de Biología, Facultad de Ciencias Universidad de Valparaíso Valparaíso Chile; ^3^ Núcleo Milenio de Salmónidos Invasores (INVASAL) Universidad de Concepción Concepción Chile; ^4^ Farallon Institute Petaluma California USA; ^5^ Instituto de Fomento Pesquero (IFOP) Valparaíso Chile; ^6^ Marine Mammal Institute Oregon State University Newport Oregon USA; ^7^ Department of Fisheries, Wildlife and Conservation Sciences Oregon State University Newport Oregon USA; ^8^ Instituto de Ciencias Marinas y Limnológicas, Facultad de Ciencias Universidad Austral de Chile Casilla, Valdivia Chile; ^9^ NGO Centro Ballena Azul Valdivia Chile; ^10^ Centro de Investigación Oceanográfica COPAS Coastal Universidad de Concepción Concepción Chile; ^11^ Centre for Ecology and Conservation University of Exeter Cornwall UK; ^12^ Institute of Marine Sciences University of California Santa Cruz Santa Cruz California USA; ^13^ Estuary & Ocean Science Center San Francisco State University Tiburon California USA

**Keywords:** Chilean coast, GPS tracking, habitat modeling, incidental capture, risk modeling, sea lions

## Abstract

Establishing marine species distributions is essential for guiding management and can be estimated by identifying potential favorable habitat at a population level and incorporating individual‐level information (e.g., movement constraints) to inform realized space use. In this research, we applied a combined modeling approach to tracking data of adult female and juvenile South American sea lions (*Otaria flavescens*; *n* = 9) from July to November 2011 to make habitat predictions for populations in northern Chile. We incorporated topographic and oceanographic predictors with sea lion locations and environmentally based pseudo‐absences in a generalized linear model for estimating population‐level distribution. For the individual approach, we used a generalized linear mixed‐effects model with a negative exponential kernel variable to quantify distance‐dependent movement from the colony. Spatial predictions from both approaches were combined in a bivariate color map to identify areas of agreement. We then used a GIS‐based risk model to characterize bycatch risk in industrial and artisanal purse‐seine fisheries based on fishing set data from scientific observers and artisanal fleet logs (2010–2015), the bivariate sea lion distribution map, and criteria ratings of interaction characteristics. Our results indicate population‐level associations with productive, shallow, low slope waters, near to river‐mouths, and with high eddy activity. Individual distribution was restricted to shallow slopes and cool waters. Variation between approaches may reflect intrinsic factors restricting use of otherwise favorable habitat; however, sample size was limited, and additional data are needed to establish the full range of individual‐level distributions. Our bycatch risk outputs identified highest risk from industrial fisheries operating nearshore (within 5 NM) and risk was lower, overall, for the artisanal fleet. This research demonstrates the potential for integrating potential and realized distribution models within a spatial risk assessment and fills a gap in knowledge on this species' distribution, providing a basis for targeting bycatch mitigation outreach and interventions.

## Graphical abstract

We applied telemetry data of a mobile and widespread species in a combined distribution modeling framework (distribution was modeled at the population‐level and individual‐level). In our northern Chile study system, the combined predictions of South American sea lions revealed unique patterns of habitat use across scales. The refined suitability map was integrated with fisheries data in a spatial bycatch risk assessment, identifying areas of greater interaction risk at the interface of industrial and artisanal fleet operational areas.

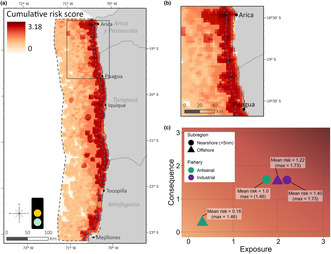

## 1 INTRODUCTION

Identifying patterns in the geographic distribution of species is a fundamental tool for management (Rodríguez et al., 2007). However, mobile marine species are challenging to observe at sea, and adequate occurrence data are often limited (Gregr & Trites, 2008). Hence, satellite tracking has become a valuable tool for collecting location data and has improved our understanding of mobile animal foraging behavior (Baylis et al., 2015; Speakman et al., 2021), habitat use (Jones et al., 2015; Reisinger et al., 2018), and anthropogenic interactions (Bedriñana‐Romano et al., 2021; Hamer et al., 2013). Telemetry data and environmental conditions can be used to create predictive habitat models which offer critical insights on spatial patterns of habitat use and can inform risk of human‐wildlife conflict (Abrahms et al., 2019; McClintock et al., 2021; Scales et al., 2016). Traditional habitat models typically estimate potential distribution, or the geographic area where a species or population could occur, providing foundational information on species‐environment relationships that can be refined and used to project future shifts (Dodson et al., 2020; Jiménez‐Valverde et al., 2008). Realized distribution, or the area a species actually occupies, is constrained by biotic interactions (e.g., competition or predation), dispersal limitations, and individual variation (Jiménez‐Valverde et al., 2008; Moudrý & Šímová, 2012).

For species with constraints on movement, such as central‐place foragers, incorporating movement limitations in habitat modeling is particularly important to reflect distance‐dependent accessibility of habitat (Matthiopoulos, 2003). Comparing distance‐constrained habitat predictions (realized distribution) with predicted environmental suitability (potential distribution) can reveal localized constraints on distribution for individuals. It can also highlight areas with potentially favorable conditions, at the regional population‐level, in places lacking survey or tracking data and inform areas of focus for conservation and management of mobile species (Moudrý & Šímová, 2012; Warwick‐Evans et al., 2018).

Despite a growing body of tracking research on mobile species, and specifically pinnipeds (sea lions, seals, and walrus) (e.g., Semenova et al., 2019; Ventura et al., 2019), the development of approaches to integrate satellite tracking data and address their complexities in the construction of correlative habitat models is an area of ongoing refinement (Northrup et al., 2022; O'Toole et al., 2021). Telemetry data are collected longitudinally for individuals and pooling of individuals in correlative models (i.e., not including a random intercept term) can be a pragmatic approach to estimate population‐level distribution patterns (e.g., Baylis et al., 2019). In contrast, mixed‐effects models can incorporate the individual as a nested variable, allowing individual‐specific predictions of spatial distribution (Briscoe et al., 2018; Fieberg et al., 2010; Koper & Manseau, 2009). Sea lions are central‐place foragers that are considered generalists at the population level, constrained primarily by resource availability and distance to terrestrial haul‐outs. However, they exhibit individual foraging specializations (Hückstädt et al., 2016; Kuhn & Costa, 2014; Lowther et al., 2011), and therefore, their realized distribution can be considered an additive function of individual‐level processes (Dodson et al., 2020; Wennekes et al., 2012). The combined habitat modeling framework described by Chambault et al. (2021) integrates potential and realized distribution approaches to create refined habitat predictions using telemetry data.

**FIGURE 1:**
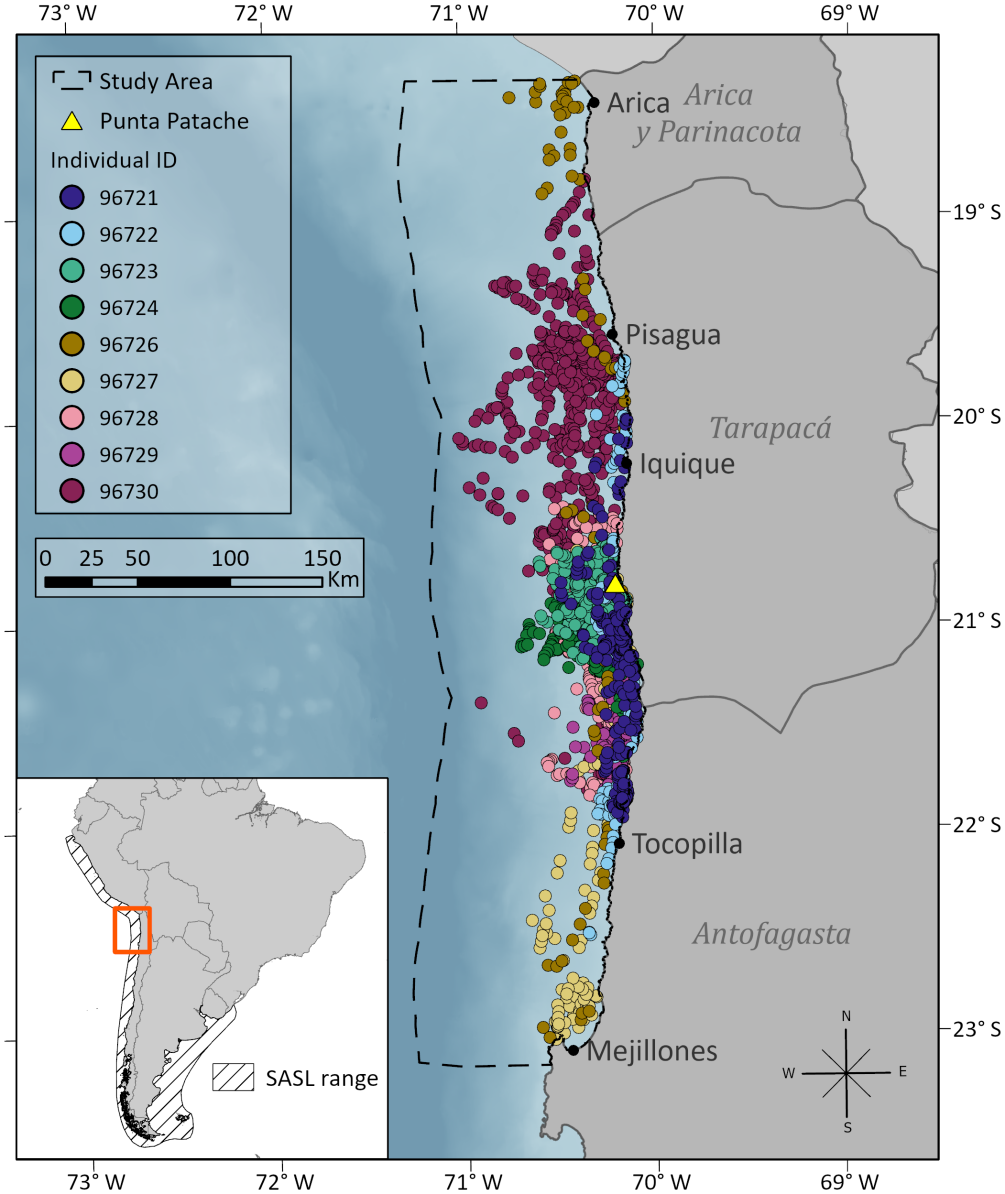
At‐sea locations of tagged South American sea lions (SASL; 6 adult females, 1 juvenile female, and 2 juvenile males) within the 100 km study area (dashed black line). The yellow triangle indicates the tagging site of Punta Patache, a significant breeding colony in the austral summer (>3000 individuals, Contreras et al., 2014).

This research extends the combined potential and realized modeling framework (Chambault et al., 2021) by integrating two models of habitat suitability in a spatial bycatch risk assessment for a population of South American sea lions (*Otaria flavescens*, SASL) in northern Chile. SASL are distributed along both the eastern Pacific and western Atlantic coasts of South America (Figure 1) and are listed as Least Concern in the IUCN Red List (Cappozzo & Perrin, 2009; Cárdenas‐Alayza et al., 2016). However, relatively little is known about their at‐sea distribution and habitat use in the Pacific (Baylis et al., 2015; Bedriñana‐Romano et al., 2014). At‐sea movement of SASL differs by age and breeding status, with juveniles limited to nearshore foraging (Hückstädt et al., 2014) and breeding females exhibiting central‐place foraging (Campagna et al., 2001; Sepúlveda et al., 2017). SASL are also responsive to environmental change (Hückstädt & Antezana, 2006; Oliva et al., 2008) and exhibit dietary plasticity following prey shifts (Muñoz et al., 2013). In northern Chile, they primarily forage on pelagic species, including some commercially harvested fish and cephalopods (Crespo et al., 2021; Muñoz et al., 2013; Sarmiento‐Devia et al., 2020).

Fisheries interactions are considered a principal threat to Chilean SASL populations and have been documented in industrial and artisanal pelagic purse‐seine fleets (Goetz et al., 2008; González et al., 2015; Hückstädt & Antezana, 2003). The artisanal fleet, which includes small and mid‐size (≤18 m) vessels (1136 total registered in 2020, Servicio Nacional de Pesca y Acuicultura de Chile, SERNAPESCA, 2020), has exclusive rights to operate within 5 nautical miles (NM) of shore (Castilla, 2010). Industrial vessels (>18 m and 50 gross tonnes; 112 registered in northern Chile, SERNAPESCA, 2020) generally operate outside the 5 NM zone but were permitted by regulation in certain nearshore artisanal areas until 2019 to maintain industry revenue (R. Vega, personal communication; e.g., Resolution Ex. N° 488–2017; SUBPESCA 2017). In northern Chile, marine productivity and fish abundance are driven by an upwelling regime from the Humboldt Current System, which is periodically disrupted by the El Niño‐Southern Oscillation (ENSO) (Morales et al., 1993; Thiel et al., 2007). Purse‐seiners primarily target anchovy (*Engraulis ringens*) but shift to target jack mackerel (*Trachurus murphyi*) under warm ENSO periods (Barber & Chavez, 1983; González et al., 2015), both of which are primary prey species of SASL in north and central Chile (Hückstädt & Antezana, 2006; Sarmiento‐Devia et al., 2020).

Conflicts between fisheries and SASL occur through indirect biological interaction, including competition for resources, and direct operational interaction, including depredation, damage to fishing gear, and incidental capture, or bycatch (Félix et al., 2021, Punt et al., 2021, Riet‐Sapriza et al. 2013). In northern Chilean purse‐seine operations, González et al. (2015) reported an average of 55 sea lions interacting per haul. Sea lion interactions in artisanal fisheries may also have considerable economic repercussions through gear damage and loss of catch (Goetz et al., 2008; Oliva et al., 2003; Sanguinetti et al., 2021; Sepúlveda et al., 2007). Under recently established import provisions, fisheries exporting to the USA are legally required to demonstrate compliance with the Marine Mammal Protection Act (MMPA). However, comprehensive monitoring and bycatch data are often lacking (Bering et al., 2022; Félix et al., 2021). Spatially explicit risk assessments are a valuable tool to direct limited management resources (Grech et al., 2008; Soykan et al., 2008). The Bycatch Risk Assessment (ByRA) toolkit, first applied in southeast Asian fisheries for Irrawaddy dolphins (*Orcaella brevirostris*) and dugongs (*Dugong dugong*; Hines et al., 2020; Verutes et al., 2020), leverages existing data and knowledge from local experts to characterize interaction risk. Local stakeholder engagement and expertise, including that of researchers, agency personnel, and fishers, is indispensable in the ByRA process to characterize the nature of interactions and compile or create data on fishing occurrence, animal distribution, and bycatch to generate risk scenarios (Verutes et al., 2020). The flexible inputs of the ByRA toolkit can accommodate limited or non‐systematic data to bridge information gaps while recognizing uncertainty, such as using fisher‐reported information in areas lacking systematic survey data (e.g., Costanza et al., 2021). ByRA scenarios can be used by agencies and stakeholders to identify and explore areas of concern for bycatch monitoring and intervention (Costanza et al., 2021; Verutes et al., 2020).

In this research, we used telemetry data of female and juvenile male SASL to model the spatial distribution of SASL in northern Chile and identify spatial patterns of risk for fisheries interactions. Using generalized additive models (GLM) and generalized linear mixed‐effects models (GLMM), we estimated population‐level (potential) and individual‐level (realized) distributions, respectively. By combining spatial predictions of the two model approaches in a single map, we identified critical areas across individual and population‐level distributions and revealed locations for future research and refinement. Finally, we used a spatially explicit risk model (Hines et al., 2020, Verutes et al., 2020) to assess interaction in industrial and artisanal purse‐seine fisheries based on fishing location data and the combined map of predicted female and juvenile male SASL distribution.

## 2 METHODS

### 2.1 Study area and animal instrumentation

The study area extends from the Mejillones Peninsula to the northernmost extent of the Chilean exclusive economic zone (18°–23° S) (Figure 1). This section of coastline is over 800 km with 18 SASL colonies recorded in 2013 (Oliva et al., 2020). This research is based on the tagging of nine SASL at the colony of Punta Patache (20°48′ S; 70°12′ W) in 2011 (Figure 1). Telemetry data were collected in July–October 2011. For details on animal capture, instrumentation, and permitting, see Hückstädt et al. (2016). Briefly, nine SASL (seven females and two juvenile males; Table S1) were captured, anesthetized, and instrumented with satellite relay data logger GPS tags (Sea Mammal Research Unit, University of St. Andrews) using marine epoxy. Tags provided high‐accuracy (<100 m for ≥6 satellites, Dujon et al., 2014) Fastloc GPS locations, and haul‐out and dive records (Costa et al., 2010). Tags were programmed to attempt a fix every 40 min.

### 2.2 Data processing

To process the telemetry data, we removed all locations calculated with <5 satellites and residual errors >35 (Dujon et al., 2014; Hazel, 2009). We filtered out points on land and any remaining erroneous locations based on visual inspection. Finally, we calculated travel speed along straight‐line tracks between locations and removed points indicating an unrealistic speed >5.5 m s^−1^ (Costa et al., 2010; Hückstädt et al., 2014).

In distribution modeling, performance is affected by the ratio of the area occupied by a species relative to the total extent of the study area, referred to as relative occurrence area (ROA) (Lobo et al., 2008). We limited the extent of analysis to 100 km from shore to balance the ROA and avoid extrapolating beyond the typical distance adult females and juveniles travel (Hückstädt et al., 2014, Riet‐Sapriza et al. 2013). All data processing and statistical analyses were done in R software (v4.1; R Core Team, 2021) and mapping in ArcGIS Pro (v2.7.1 Esri; Redlands, CA, USA).

### 2.3 Habitat modeling

Following the framework of Chambault et al. (2021), we developed two modeling approaches: (1) a generalized linear model (GLM; binomial distribution), fitted with pooled occurrences, to predict population‐level suitability, and (2) a generalized linear mixed‐effects model (GLMM; binomial distribution), with individual ID as a random effect variable, to predict individual‐level and realized distribution (Jiménez‐Valverde et al., 2008). To avoid pseudoreplication, we spatially rarefied occurrences, retaining one presence per 1 km grid cell in the GLM and one grid cell presence per individual in the GLMM (Hirzel et al., 2006). We also tested a reduced occurrence dataset (30 locations per individual; 90% reduction) to eliminate imbalance in individual sampling and reduce serial autocorrelation; results were compared to the grid‐sampled data model (SI 2.4.2).

#### 2.3.1 Habitat variables

We selected eight environmental predictor variables as proxies for biological productivity, and to represent topographic variation and constraints of SASL movement (Baylis et al., 2019; Campagna et al., 2001) (Table S1). Oceanographic variables included mean sea surface temperature (SST;°C), chlorophyll‐α (Chl; mg m^−3^), and eddy kinetic energy (EKE; m^2^ s^−2^). SST was obtained from the Multi‐Scale High Resolution Analysis database (MUR Level 4 SST; Chin et al., 2017) and Chl was from the ESA Ocean Color Climate Change Initiative (Sathyendranath et al., 2020). Both SST and Chl were averaged for the entire study period because of monthly‐level gaps and low within‐season variability (Ancapichún & Garcés‐Vargas, 2015; Morales et al., 1993). EKE was derived from sea surface height anomalies (MEaSUREs Gridded Sea Surface Height Anomalies; Zlotnicki et al., 2019) as a measure of mesoscale activity (Baylis et al., 2019; Briscoe et al., 2018). For nearshore areas with gaps in SST, Chl, and EKE (<3% of the study area), we used a focal neighborhood mean (circle, 2 cell radius) to fill missing values (Bonhomme et al. 2007; Hogrefe et al., 2008). Although gap‐filling introduces uncertainty and does not account for nearshore variability, this maintains the original range of values and avoids extrapolation.

Topographic variables included seafloor depth (GEBCO Compilation Group, 2021; m) and four GIS‐derived layers: seafloor slope (degrees), Euclidean distance to shore (m), distance to continental shelf (m), and distance to river mouths (m). All variables were sampled (bilinear interpolation) to the 1 km^2^ resolution of the SST grid to best match the fine scale of the telemetry data. We examined the distribution of variables and used a logarithm transformation for both Chl and depth, applying a square root transformation to slope. We rescaled variables (0–1) and tested all for collinearity using Pearson's correlation coefficients and variance inflation factors (Marquaridt, 1970) prior to model fitting and removed distance to shelf and distance to shore variables due to high correlation (Pearson's *r* ≥ 0.7 or VIF > 4.5; Figure S1).

#### 2.3.2 Pseudo‐absence generation

We used an environmental dissimilarity approach to generate pseudo‐absences for each modeling approach (Senay et al., 2013), based on the assumption that true absences are more likely to occur in environmentally different locations than presences (Hazen et al., 2021). The proportion of pseudo‐absences can positively or negatively impact model performance (Barbet‐Massin et al., 2012; Hysen et al., 2022). We used the R package mopa (Iturbide et al., 2015) to create pseudo‐absence points in equal proportion to presences to balance representation in the dataset, given the wide distribution of occurrences (Figure 1; Senay et al., 2013). First, the environmental background grid of the study area was classified as presence or absence using a presence‐only profiling algorithm: one‐class support vector machines (OCSVMprofiling, from *mopa*) (Drake et al. 2006, Iturbide et al., 2018, Senay et al., 2013). For the population approach, pseudo‐absences were randomly created outside a 5 km buffer of presence points and within the “absence” area classified by the OCSVM. This approach aims to be representative of conditions where individuals could have occurred but did not, approximating environmental absences, or absences due to a lack of environmentally favorable conditions (Lobo et al., 2010).

For assessing realized distribution, absences should be located nearer to presences and reflect potentially habitable areas which are not occupied due to other biotic factors such as interactions and inter‐individual variability (Lobo et al., 2010). Therefore, in the individual approach, we limited the background extent for pseudo‐absences to a convex hull of all presences and used a smaller buffer around presence points of 2 km, twice the size of our covariates. In this approach, environmental profiling and pseudo‐absences were created individually. The resulting pseudo‐absences are environmentally dissimilar to presences and are more distant from presences in the population approach than in the individual approach (SI 2.2; Barbet‐Massin et al., 2012).

#### 2.3.3 Population model approach

We fit binomial GLMs (logit link function) with the grid sampled SASL telemetry locations, with a response variable of presence (1) versus absence (0: pseudo‐absence). To improve spatial independence and the accuracy of model performance estimates, we partitioned the data into training and test sets for cross‐validation using spatial blocking (Roberts et al., 2017). A block size of 60 km^2^ was defined by the median range of autocorrelation of the environmental data, estimated from variograms (R package *blockCV*; Valavi et al., 2019; SI 2.3). Blocks were defined across the study area and assigned to one of *k* = 5 folds for cross‐validation. We iterated (*n* = 100) the random assignment of blocks to folds to achieve an even dispersal of presences and absences (Robinson et al., 2021). Full GLMs were fitted with eight environmental predictor variables: SST, Chl, EKE, depth, slope, distance to coast, distance to shelf, and distance to river mouths.

#### 2.3.4 Individual model approach

We fit binomial GLMMs (logit link function) with the individual‐based pseudo‐absences to predict realized distribution (R package *lme4*; Bates et al., 2015; Chambault et al., 2021; Koper & Manseau, 2012). Individual ID was included as a random effect variable, allowing for differences in the baseline likelihood of presence to vary across individuals (Chambault et al., 2021, Koper & Manseau, 2012). As a measure of dispersal and central‐place constraint, we included the probability surface raster from a negative exponential kernel (NEK) in the GLMM, which represents the rate of decline in probability of individual occurrence moving away from the tagging site. A parameter *a* (range 0–1) controls how quickly the probability decreases with distance, with low values modeling limited movement, and higher values modeling long‐distance movement (Meentemeyer et al., 2008). We selected an optimal value of *a* by testing a logarithmic sequence of 10 potential values for each individual in the GLMMs (Hattab et al., 2017). The final value of *a* was selected based on model predictive performance, measured by the continuous Boyce index (Boyce., 2006; Chambault et al., 2021; Hirzel et al., 2006; Meentemeyer et al., 2008). To assess the ability of the GLMM to predict novel individuals, cross‐validation was done by leaving out one individual as a test dataset for each run (9 individuals, *k* = 9) (Koper & Manseau, 2012; Raymond et al., 2015).

#### 2.3.5 Model selection and evaluation

We evaluated model performance using averaged metrics of the test datasets from each cross‐validation run. Metrics included goodness‐of‐fit (*R* ^2^, conditional and marginal for the GLMM), sensitivity, specificity, true skill statistics (TSS; Allouche et al., 2006; Hazen et al., 2021), area under the receiver operator curve (AUC), and the Continuous Boyce Index (CBI) (R package *ecospat*; Di Cola et al., 2017). We used AUC, a common metric for comparing model parameterizations, to guide variable selection through backward elimination (Jiménez‐Valverde et al., 2013; Lobo et al., 2008). CBI indicates a model's ability to predict presences and was used to evaluate predictive performance (Hirzel et al., 2006). We examined spatial autocorrelation of model residuals by constructing variograms (R package *gstat*; Pebesma et al., 2015; Veloz, 2009).

#### 2.3.6 Spatial suitability predictions

Using the final population‐level model (GLM), we created a population‐level distribution prediction for the study period (July–October) showing the probability of female and juvenile male SASL occurrence, scaled 0–1. Using the final individual‐level model (GLMM), we created an average of the conditional (individual) predictions. The imbalanced distribution of occurrences due to tagging location and inter‐individual variability may contribute to underestimation of suitability. As a precautionary approach for the risk assessment, we created a maximum individual probability raster, using the highest predicted suitability across individuals for each cell. Finally, we combined the two model predictions by plotting the population prediction versus the maximum individual prediction raster to visualize agreement and disagreement (Chambault et al., 2021). Using quantiles, the final geospatial layer of the combined distributions was classified as high, medium, or low overall suitability based on the recommendations in Chambault et al. (2021), and modified for our system to emphasize areas with high potential distribution (Figure S15). The classified suitability layer was then used as input into the bycatch risk assessment.

### 2.4 Bycatch risk assessment

We used a spatially explicit Bycatch Risk Assessment (ByRA) model (Hines et al., 2020; Verutes et al., 2020) to identify interaction risk for SASL in Chilean northern pelagic purse‐seine fisheries. The ByRA model uses the Habitat Risk Assessment tool in the open‐source model suite InVEST (v3.12; Sharp et al., 2020), from the Stanford Natural Capital Project (naturalcapitalproject.stanford.edu/software/invest). In the ByRA framework, risk is assessed using two dimensions of information: “exposure” to a stressor and “consequence,” of exposure (Arkema et al., 2014; Samhouri & Levin, 2012; Sharp et al., 2020). For a detailed description of risk calculation, see Sharp et al. (2020). In short, a flexible set of criteria is scored within an exposure and consequence matrix, and risk is calculated as the Euclidean distance to the origin within exposure‐consequence space (Samhouri & Levin, 2012). The ByRA model uses a suite of spatial and non‐spatial scoring criteria, specifically designed to address characteristics of fishing activity, the species of interest, and properties of interaction that influence bycatch risk. The criteria are populated in a template scoring table for use in the Habitat Risk Assessment tool and risk is calculated as a continuous raster.

#### 2.4.1 Exposure and consequence criteria

Exposure criteria represent the degree to which a population experiences a stressor, given management practices. Consequence criteria describe species‐specific resilience and sensitivity to stressors (Arkema et al., 2014; Hines et al., 2020). Criteria are numerically scored: low (1), medium (2), high (3), or null (0) to omit a criterion. Final risk values are the weighted average of the criteria score, a data quality score, and an importance weighting score (Arkema et al., 2014; Verutes et al., 2020), allowing better quality and high importance criteria to have greater influence in risk calculation (Arkema et al., 2014). We followed the criteria definitions and rating scheme from previous applications of ByRA, which produces a maximum risk score for a single stressor of 2.83 (maximum of 5.66 for two overlapping stressors; Costanza et al., 2021; Hines et al., 2020; Verutes et al., 2020). Non‐spatial exposure criteria included: (1) temporal overlap between gear and species, (2) catchability in gear, and (3) current status of management action. Consequence criteria incorporate life history traits and population dynamics; these included: (1) age of maturity for the species, (2) reproductive strategy, (3) population connectivity, (4) local species conservation status, (5) mortality from gear, and (6) life stages affected by gear (Verutes et al., 2020). All criteria, data quality, and weighting scores were decided based on a review of previous literature and expert input from all co‐authors (SI 3.1).

#### 2.4.2 Spatially explicit criteria

We divided the study area into two subregions to evaluate risk: the nearshore, predominantly artisanal fishing zone (<5 NM), and the offshore region. Spatially explicit criteria (SEC) were incorporated in the exposure‐consequence scoring table by inputting a file path to a pre‐formatted, geospatial layer, with values re‐classified to match the scoring schema (Costanza et al., 2021; Sharp et al., 2020). We used three SEC in the ByRA model:

1. Intensity of fishing activity: calculated using fishing set location data (2010–2015) from the Fisheries Development Institute's (Instituto de Fomento Pesquero; IFOP) monitoring program of pelagic fisheries of northern Chile. Industrial fleet data were recorded by onboard scientific observers (4%–5% annual coverage). For the artisanal fleet, fishing points from shipowners' reporting had been assigned to a spatial grid of 1 × 1 nautical miles. To estimate intensity of gear use (i.e., relative density of fishing sets), given geographic uncertainty, we ran kernel density estimations with a resolution of 3 km (5 km search radius) (Shahrabi & Pelot, 2009). For input into ByRA, the continuous relative density values were reclassified (1–3) by quantiles (Figure S14).
2. Overlap between species and fishing activity: evaluated automatically by the HRA tool as the spatial overlap (cell‐by‐cell) between species distribution and fishing intensity, where cells with overlap receive a risk score calculation and those without receive a score of 0 (Sharp et al., 2020; Verutes et al., 2020). For the species distribution, we reclassified the combined suitability map to emphasize agreement between the models (Figure S15).
3. Likelihood of interaction: calculated from the sum (cell‐by‐cell) of the fishing intensity SEC and the combined map of SASL distribution, reclassified similarly (Hines et al., 2020) (Figure S16).

#### 2.4.3 Assessing uncertainty

We used a diagnostic matrix (Table S3) to characterize the uncertainty of four types of data used in ByRA: (1) animal presence data, (2) habitat suitability estimation, (3) fishing effort data, and (4) available bycatch data (Hines et al., 2020). Uncertainty is visualized with a stoplight framework of green, yellow, and red, indicating low to high uncertainty. Output risk maps are presented with a stoplight icon to highlight data limitations (Verutes et al., 2020).

## 3 RESULTS

After processing, telemetry data from six adult females, one juvenile female, and two juvenile males SASL yielded 2660 locations. Filtering removed 533 locations and an additional 9 were located outside the study area and excluded, based on the ROA. Tag deployment duration ranged from 31 to 125 days (Table S1). All animals remained north of the Mejillones Peninsula throughout the tag deployment period (Figure 1) and used multiple terrestrial haul‐out sites (mean 5.78 ± 3.72).

### 3.1 Habitat models

#### 3.1.1 Population model

**FIGURE 2:**
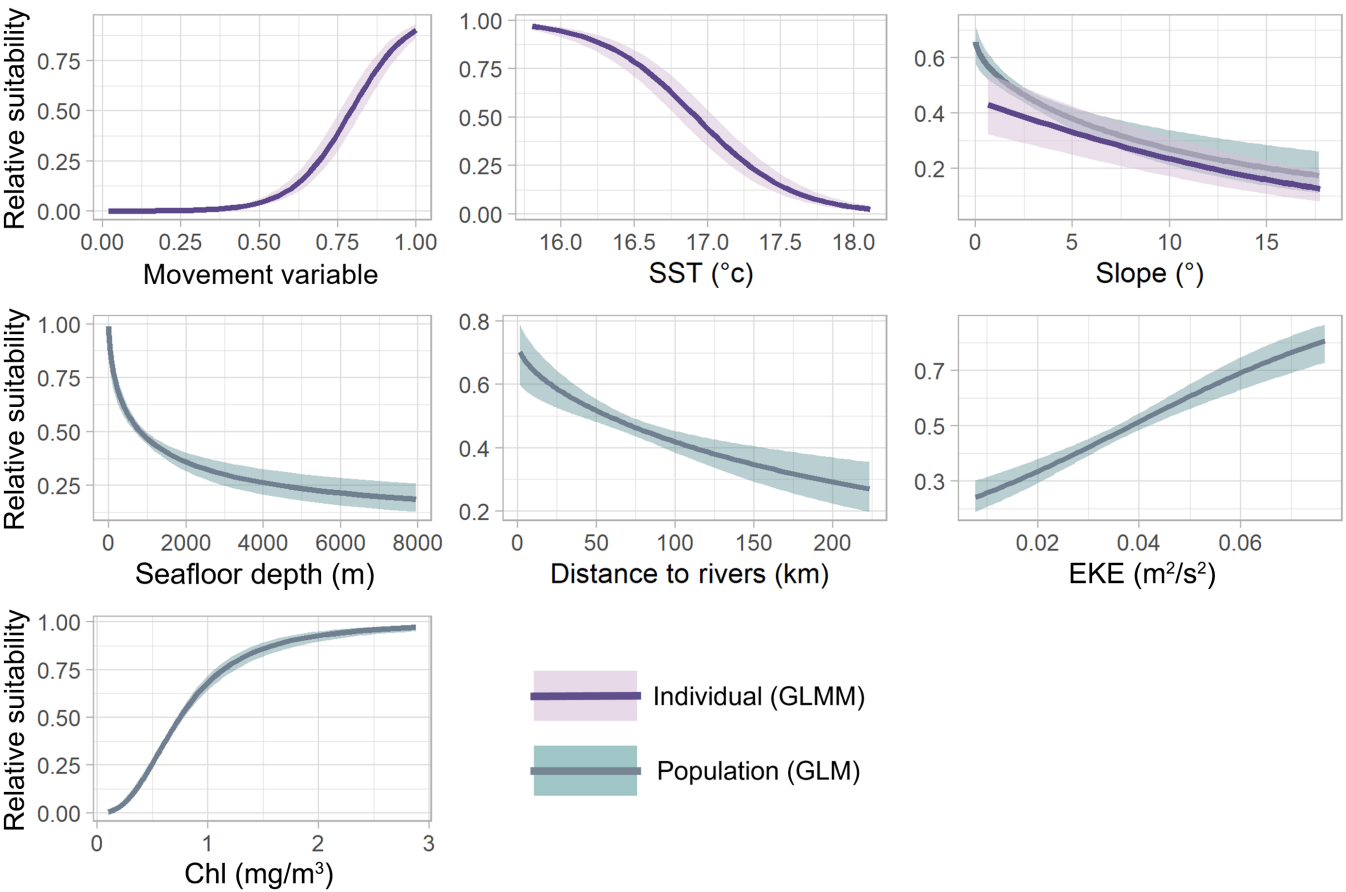
Individual (GLMM) and population (GLM) model response plots showing the relationship of relative habitat suitability for female and juvenile SASL with seven variables: Negative exponential kernel (NEK) movement variable, sea surface temperature (SST), slope, seafloor depth, distance to rivers, eddy kinetic energy (EKE), and chlorophyll‐α (Chl). 95% confidence intervals are shown in shaded bands.

The final GLM included five variables which significantly influenced population‐level habitat suitability of female and juvenile male SASL: Chl, slope, depth, distance to rivers, and EKE. Distance to shelf and distance to shore were both removed before model fitting due to high correlation with multiple other variables (Pearson's correlation coefficient ≥ 0.75, Figure S1). The model had a high mean CBI across runs (Table 1), indicating suitability predictions which are consistent with presences in the test data. Chl was the most important predictor, as determined by the absolute t‐statistic for each variable (Figure S13). Partial response curves (Figure 2) showed highest population‐level suitability for Chl values over 2 mg/m^3^, and depth below 1000 m. We identified a positive relationship between SASL occurrence and EKE, and a negative relationship with increasing distance from rivers (Figure 2). The model built with a reduced dataset had lower performance metrics overall (mean CBI: 0.86 ± 0.14; Figure S5) and did not meaningfully decrease residual spatial autocorrelation (SI 2.5.1).

**TABLE 1:** Final predictors for the full and reduced (red.) datasets in the population (negative binomial GLM) and individual (negative binomial GLMM) model approaches with mean (±SD) area under the receiver operating curve (AUC) and Continuous Boyce Index (CBI) values.

Model	Predictors	Mean AUC	Mean CBI
Full population	1/0 ~ EKE + depth + slope + Chl + EucDistRivers	0.95 ± 0.03	0.96 ± 0.02
Red. population	1/0 ~ EKE + depth + slope + Chl + EucDistRivers	0.95 ± 0.03	0.81 ± 0.14
Full individual	1/0 ~ SST + NEK + slope + (1|ID)	0.93 ± 0.09	0.84 ± 0.15
Red. individual	1/0 ~ SST + NEK + (1|ID)	0.92 ± 0.09	0.69 ± 0.17

*Note*: Variables include eddy kinetic energy (EKE), seafloor depth (depth), seafloor slope, chlorophyll‐a (Chl), distance to rivers (EucDistRivers), and the negative exponential kernel (NEK).

#### 3.1.2 Individual model

The optimal coefficient of the negative exponential kernel (NEK)t in the individual model, which maximized CBI across folds, was 0.04. The final model included the NEK, SST, and slope. The mean conditional *R* ^2^ (variance explained by random and fixed effects) and mean CBI suggested a well‐fitted model (Table 1). Response plots for the GLMM showed a negative relationship between individual occurrence and increasing SST (Figure 2). The relationship to slope showed a similar pattern in the individual and population models, with a larger confidence interval in the individual model. The model built with a reduced dataset did not perform as well and had a larger range of autocorrelation (Figures S6 and S10).

#### 3.1.3 Suitability predictions

**FIGURE 3:**
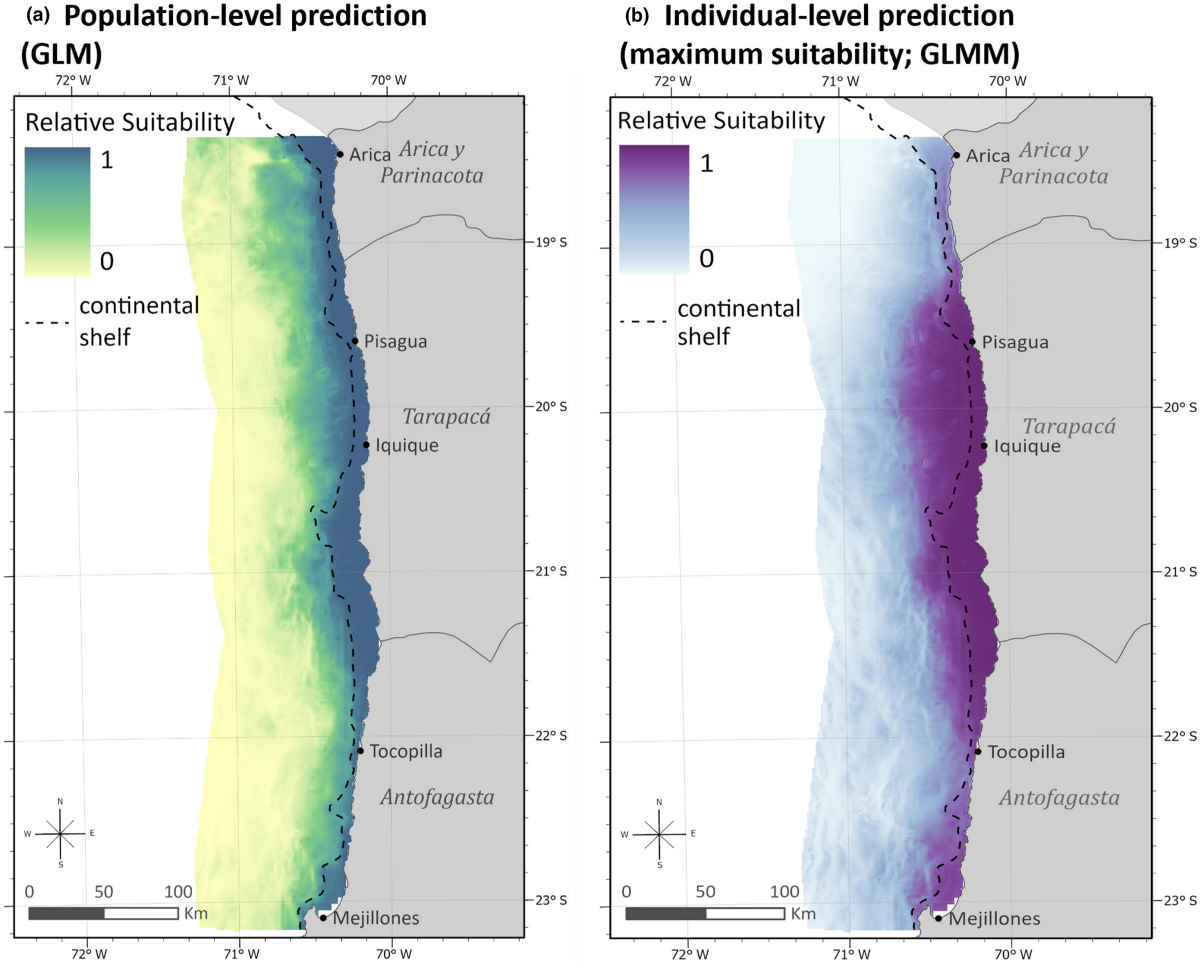
Spatial suitability predictions (scaled 0–1) for the population (a) and maximum individual (b) models. The continental shelf boundary is shown as a dashed black line, as defined by the 200 m depth contour.

The population‐level distribution prediction highlighted broad coastal suitability, with a divergence along the shelf break (Figure 3a). Moderate suitability extended further off the coast in the far north, near the shelf break in Peru. The lowest population‐level suitability was in offshore areas, past 50 km. The individual distribution prediction, based on maximum probabilities, highlighted moderate suitability further offshore, from approximately Tocopilla to Pisagua as well as near Mejillones Bay (Figure 3b). The area north of Pisagua, which had more absences than presences, showed very low individual‐level suitability.

**FIGURE 4:**
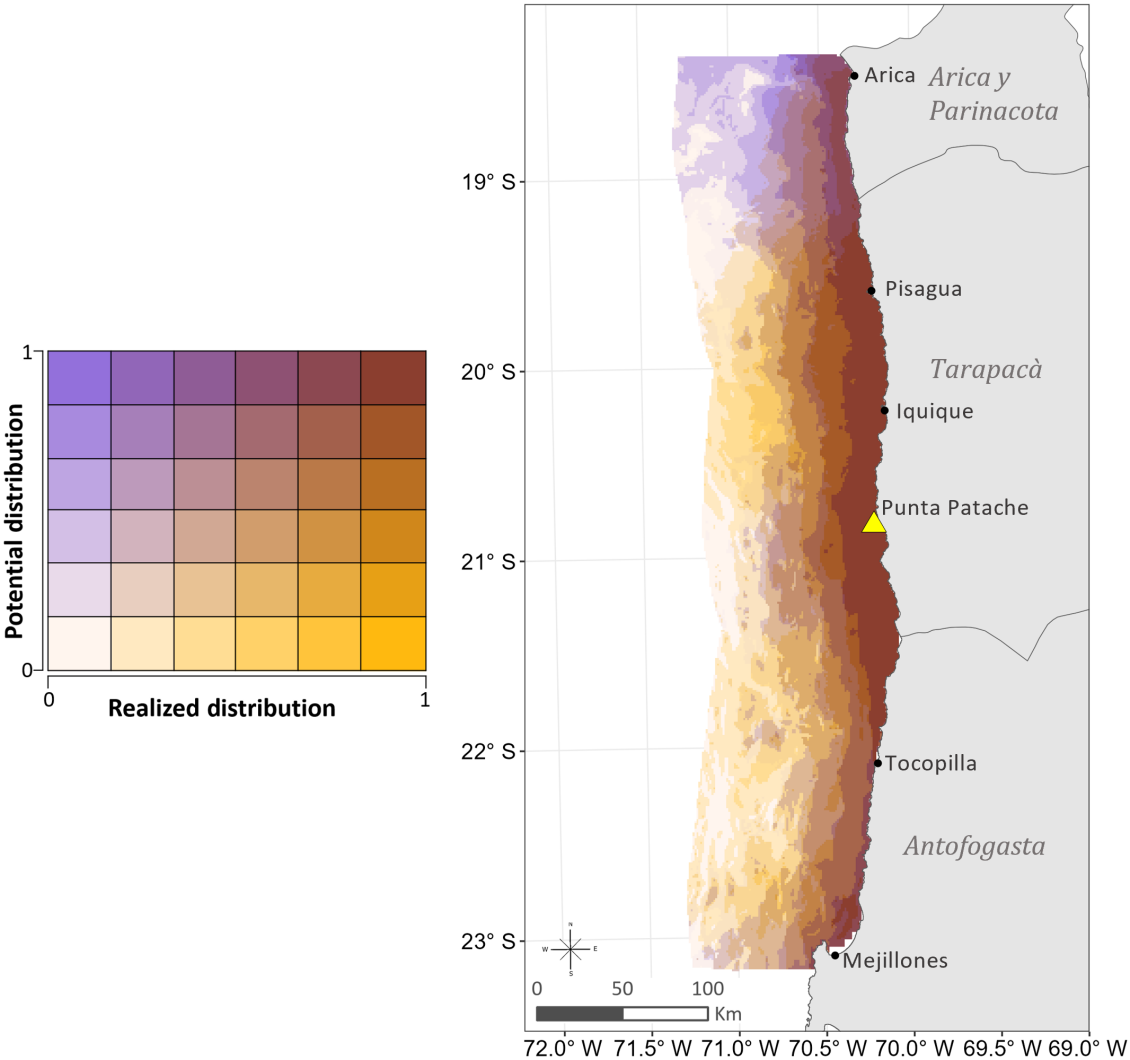
Plot of the combined potential (population‐level) versus realized (averaged individual‐level) distribution predictions in three‐dimensional color space. Purple areas indicate high suitability at the population‐level for females and juvenile male SASL. Yellow areas represent greater likelihood of individual SASL occurrence. The brown color shows areas of high likelihood of occurrence at both the population and individual levels, illustrating high agreement between the potential and realized distributions. Low suitability for both models is shown in off‐white.

The bivariate color map, combining potential and realized distributions, reflected large areas of agreement between models within approximately 25 km of shore along the coast (Figure 4). Areas north and south of the tagging site, approximately 22° S–19.5° S, had very high overlap (brown areas), while the lowest suitability for both models (off‐white) was offshore, south of 20.5° S. Areas of higher realized probabilities also occurred offshore, specifically between Iquique and Pisagua. High potential probabilities were largely in the north (purple), where there were fewer individual occurrences.

### 3.2 Bycatch risk assessment

#### 3.2.1 Uncertainty

**FIGURE 5:**
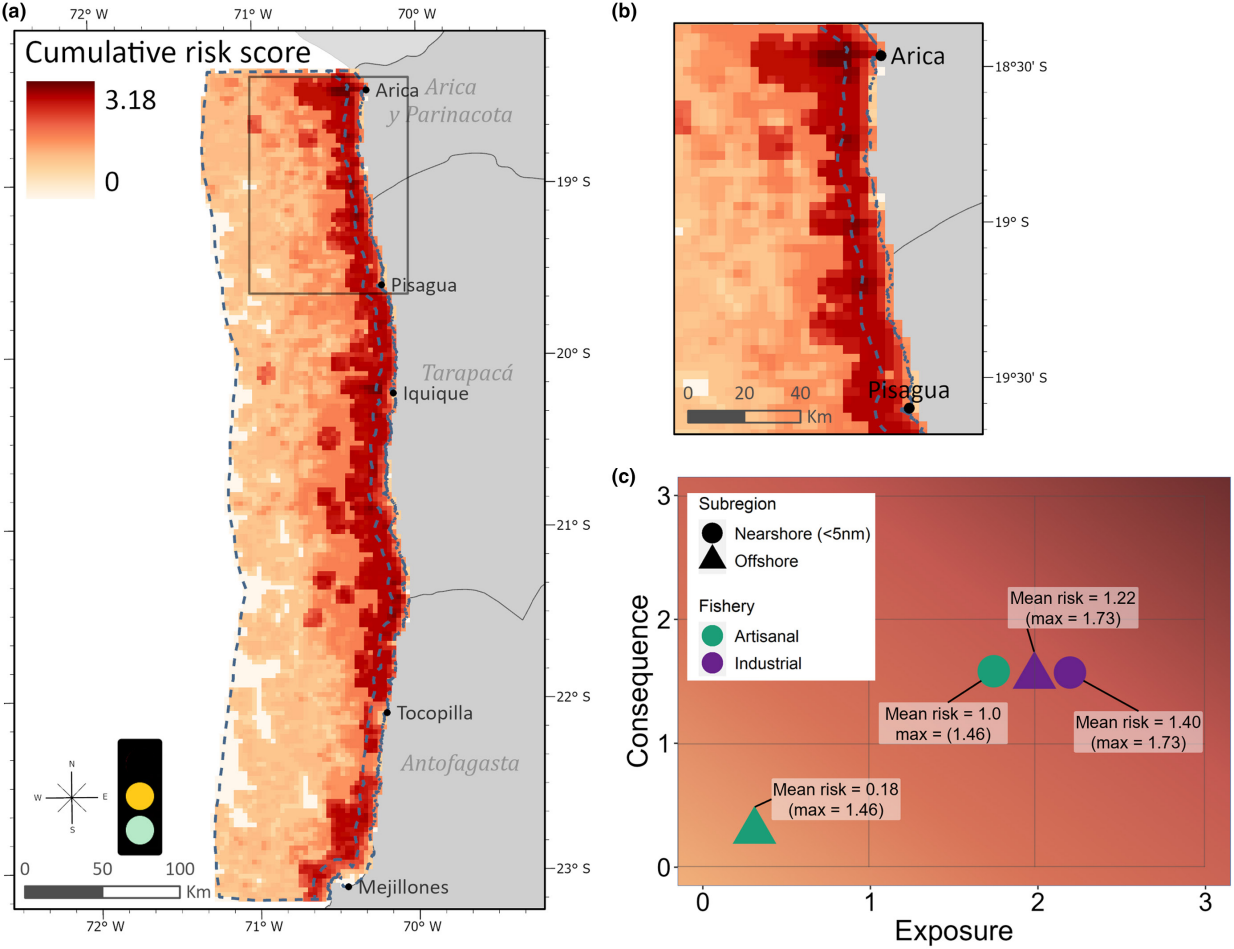
(a) Cumulative estimated bycatch risk (sum of both fisheries, score 0–6) for female and juvenile South American sea lions in industrial and artisanal purse‐seine fisheries, in austral winter and early spring (July–October). Subregions (dashed blue line) include the nearshore fishing zone (within 5 nm) and offshore zone (up to 100 km). Moderate data uncertainty is indicated by the stoplight and described further in Results, with lower uncertainty (green) associated with the animal occurrence and habitat suitability data and medium uncertainty (yellow) for fishing occurrence and bycatch information. (b) Inset of the northernmost study area shows details of risk along the subregion boundary. (c) The exposure and consequence plot depicts the mean and maximum risk scores for each fishery, by subregion, as a function of exposure and consequence scores (Table S2).

Based on the standards in Verutes et al. (2020), we ranked both the animal occurrence and habitat suitability data as low uncertainty (green stoplight; Figure 5a) because of the quality of the telemetry data and performance of the habitat models (Table S3). Fishing occurrence was assigned medium uncertainty (yellow; Figure 5a), as we used observer data for the industrial fleet (annual coverage 4%–5%) and spatially aggregated fisheries logbook data for the artisanal fleet, where observer data were unavailable. Bycatch information was designated as medium uncertainty, as no data were available to include in this research; however, mortality rates with SASL in northern purse‐seine fisheries have been estimated in González et al. (2015).

#### 3.2.2 Risk estimates

Risk assessment outputs include geospatial layers of cumulative risk, the cell‐by‐cell sum of risk from both fisheries (Figure 5a,b), and plots of risk within exposure‐consequence space (Figure 5c). Most higher risk areas followed the spatial overlap between industrial and artisanal fishing, along the border between the nearshore and offshore subregions. The risk map showed highest risk in the northern regions of Arica and Parinacota, with another focal area near the city of Iquique (Figure 5a,b). Risk diminished with distance from the coast, and the southern part of the study area showed relatively low risk overall.

Exposure and consequence plots indicated the highest risk for both industrial and artisanal fishing activity was in the nearshore subregion, where habitat suitability was highest (Figure 5c). Nearly 20% of the industrial fishing set locations were within the nearshore zone and the highest risk was in that subregion. The Euclidean distance‐based risk calculation produces a maximum potential risk score for a single stressor of 2.83. The maximum cumulative risk in the nearshore subregion (summed maximum risk from both fisheries) was 3.18. A low number of artisanal fishing sets occurred in the offshore region, corresponding to a low mean risk there (0.17 artisanal, 1.22 industrial). Across both subregions, the maximum risk for the artisanal fishery was 1.4 and for industrial fishery 1.73.

## 4 DISCUSSION

### 4.1 Combined model approach

Using telemetry data of adult female and juvenile SASL, we developed a distribution map combining population‐level and individual‐level habitat models. This map highlights habitat that is likely to be occupied near the tagging site, as well as revealing potentially suitable areas further north and south of the sampled colony. Our population model (GLM) aimed to illustrate potential distribution by identifying predicted areas of environmental suitability for females and juvenile sea lions throughout the study area. The individual‐level model, targeting realized distribution, incorporated individual movement constraints and was more strongly influenced by tagging site bias. Comparing the model built with only environmental variables against the individual model highlights areas of divergence and agreement, including areas where individuals may occur beyond what is predicted by the population‐level model and vice versa. Critically, this combined approach allowed us to incorporate potential areas of suitability, beyond the distance‐constrained individual‐level prediction near the tagging colony, in order to develop an initial bycatch risk assessment for the target population in northern Chile. Given the economic and sociocultural importance of these fisheries and the abundance of SASL in northern Chile (29,896 individuals estimated in the three northernmost administrative regions in 2020; Oliva et al., 2020), evaluating spatial trends in bycatch risk, even in data‐limited scenarios, enables scientists and managers to characterize threat across fisheries and inform bycatch prevention and reduction measures (Grech et al., 2008; Punt et al., 2021).

### 4.2 Ecological implications

Sampled individuals made use of multiple haul out sites during the tracking period, indicating extended movements apart from the colony at Punta Patache. While the individual model is most relevant near the tagging site, the population model integrates environmental suitability for individuals from other colonies in the study area into a combined map, providing a preliminary evaluation of distribution for the region.

The models support previous findings on the association between SASL occurrence and upwelling indicators, Chl and SST, and greater distribution of females and juveniles within 75 km of shore (Campagna et al., 2001; Hückstädt et al., 2014; Soto et al., 2006). However, caution is needed in interpreting and generalizing model results based on nine individuals during a non‐breeding season, given the dynamic environment and inter‐individual foraging plasticity (Grandi et al. 2021, Hückstädt et al., 2016). Additionally, while the geographically and environmentally separated pseudo‐absence sampling approach provided greater specificity in the model predictions it also may have contributed to increasing the performance metrics for both models (Hazen et al., 2021).

The distinct modeling procedures likely influenced the division of SST and Chl between model types. SST reveals thermal regimes, which directly influence anchovy dispersion (Gutiérrez et al., 2007; Silva et al., 2019), while Chl reflects primary productivity, which has a lagged effect on upper trophic levels (Montalva et al., 2022). Both variables influence prey abundance and aggregation in this productive upwelling system (Fadely et al., 2005; Kuhn & Costa, 2014); however, the Chl satellite imagery had a coarser native resolution of 4 km^2^ (Table S2), so we were unable to detect fine‐scale oceanographic dynamics. The importance of mesoscale eddies, represented by EKE, parallels the findings of Briscoe et al. (2018) for California sea lions (*Zalophus californianus*) in the California Current System. In northern Chile, coastal eddies can promote recirculation of plankton, influencing fish recruitment (Palma et al., 2006; Rojas & Landaeta, 2014). Additionally, physical gradients between eddies and the surrounding water mass can support aggregation of prey species (Bailleul et al., 2010; Kai & Marsac, 2010).

While slope was significant in both model types, the trend was relatively weak, likely due to the shallow slope across much of the study area (mean 4.5° ± 3.5), with steeper values offshore where SASL occurrence was notably lower. NEK, the distance‐based movement variable, in the individual model additionally captured movement constraints, which are fundamental to estimating realized distribution (Chambault et al. 2021; Lobo et al., 2006). The high individual suitability at latitudes south of 19° S (Figure 3b), in part, reflects bias from greater occurrences in proximity to the tagging location. However, this region also exhibits year‐round upwelling and includes the Loa River mouth at 21.4° S (Fonseca & Farías, 1987; Palma et al., 2006; Thiel et al., 2007). The river mouth area is associated with spawning grounds for anchovy, a primary prey item for SASL in the north (Herrera & Escribano, 2006; Palma et al., 2006; Sarmiento‐Devia et al., 2020). Prey aggregation largely shapes marine habitat of pinnipeds; however, the individual model underscores the influence of habitat accessibility in determining foraging habitat.

### 4.3 Management relevance

Motivated, in part, by the Seafood Import Provision in the USA (Félix et al., 2021), increasing efforts have been made to monitor bycatch in Chile through scientific observer programs, led by the IFOP. However, observer coverage is generally low in pelagic purse‐seine fisheries, especially for the artisanal fleet in northern Chile (R. Vega, personal communication). As fisheries management works toward compliance with the import provision, new mitigation measures are being established and there is a need for prioritization and targeting of management.

SASL are behaviorally attracted to fishing operations and niche overlap occurs as fishers and SASL target the same marine resources (de Oliveira et al., 2020; Hückstädt & Antezana, 2003). Our ByRA model produced maps of interaction risk, which can be classified (low, medium, and high) for easy interpretation by stakeholders. Cumulative risk from both fleets was moderate overall but the greatest risk areas occur near the boundary of the 5 NM nearshore artisanal zone (Figure 5). This follows the trend of high combined distribution of SASL in the nearshore (Figure 5).

Our integration of the combined distribution map in ByRA provided specificity by refining areas of high potential suitability for the population (females and juveniles) with predictions of individual‐level occurrence, or realized distribution. While our risk results are most relevant to females and juveniles, the higher industrial fishing risk in the nearshore region calls attention to the threat of the industrial fleet operating in higher consequence, nearshore areas. However, the pattern observed in this study may change significantly as industrial fleet access to the artisanal fishing zone has been restricted since 2021 (Res. Ex. N° 1238–2021; SUBPESCA 2021). Moderate risk for nearshore artisanal fishing should also be noted, as any interaction with small‐scale fishing can negatively impact both the animals and fishermen directly (Davis et al., 2021). Conflict at the small‐scale also affects fisher's attitudes toward the species, potentially influencing the efficacy of conservation measures (Pont et al., 2016).

Harm reduction regulations (e.g., SUBPESCA Res. Ex. N° 2667–2021) and incidental capture reporting requirements can reduce mortality events and increase awareness of bycatch events; however, a broader goal of bycatch prevention should be upheld. The ByRA framework allows for multiple scenarios of fishery and animal distribution to be investigated, which stimulates stakeholder engagement through review and discussion of outputs (Costanza et al., 2021; Hines et al., 2020). The strong negative influence of El Niño events and marine heatwaves on coastal fisheries resources (Hernández‐Santoro et al., 2019; Lehodey et al., 2020), combined with greater regulations and monitoring have contributed to a reduced size of the industrial purse‐seine fleet in northern Chile (Armas et al., 2022; Böhm et al., 2017). Future ByRA scenarios should aim to model recent fishing effort, specifically under El Niño conditions, and explore the potential higher consequence of interaction for SASL with fisheries, given potential shifts in foraging conditions from altered prey distribution (Riet‐Sapriza et al. 2013, Hückstädt & Antezana, 2006).

### 4.4 Study limitations

As with any model, our habitat predictions should be regarded as provisional and subject to improvement, particularly given the sampling and behavioral biases inherent to the animal and fishing occurrence data. Our tagging sample was limited to nine females and juvenile animals, which exhibit constrained movements relative to adult male SASL (Goetz et al., 2008; Sanguinetti et al., 2021). The small sample size does not necessarily represent the full spectrum of habitat use and movement patterns within the target population and makes generalizing the model findings challenging. In combination with the environmentally and spatially stratified approach to pseudo‐absences, the small sample may have contributed to slight model overfit and optimistic performance metrics, although this was not detected in the spatial cross‐validation procedure. Additionally, the population model likely underestimates potential distribution, and consequently bycatch risk, in this region due to the use of individual occurrence data from a single colony. Despite this restricted scope of inference from a narrow population sample, our models effectively utilize available telemetry data to provide an initial evaluation of habitat correlations and interaction risk and the novel methodology may be applicable to similar mobile species.

Spatial autocorrelation is a typical property of wildlife movement data that could not be entirely removed in the models. Additionally, some autocorrelation of the environmental variables can be attributed to having re‐sampled these data to a finer scale than the native resolution. Fieberg (2007) suggests that downsampling occurrences to try and reach independence may be counterproductive, instead placing emphasis on representativeness. The supplemental models, developed using a 90% reduced dataset did not have a notable effect on reducing the range of residual autocorrelation, as indicated by variograms, and were not considered representative (SI 2.5.2). The spatial cross‐validation procedure of the population approach and random effects structure in the individual approach were considered robust for our purpose of predicting distribution within a set study region (Fieberg et al., 2010; Koper & Manseau, 2009).

Scientific observer data with low trip coverage (<5% annual coverage) and geographic uncertainty in the artisanal fishing logs provided a limited representation of industrial fishing activity in the region. However, our fishing distribution maps showed well‐dispersed activity within the study area, indicating adequate coverage at the scale of 4 km^2^ (Figure S14).

### 4.5 Future directions

Future work should aim to collect location data from representative age and sex classes in the population, including adult males. Although tagging of adult males is logistically infeasible, visual line‐transect surveys such as Bedriñana‐Romano et al. (2014) can provide valuable at‐sea data for an entire population. This might be evaluated by incorporating observations of SASL in ongoing cetacean surveys off the Chilean portion of the Humboldt Current ecosystem (Bedriñana‐Romano et al., 2022). The combined distribution map illustrates areas of environmental suitability, based on the population‐level model, north of the tagging site. Surveys or tagging efforts in these areas could be used to validate and refine these predictions.

Our results support the idea that upwelling processes have a strong influence on SASL distribution patterns. Because this relationship is highly dynamic and undergoing shifts from climate change, effort should be made to examine the role of SST fronts as well as environmental associations under multiple spatiotemporal scales. Such analyses could evaluate weekly variability in relation to upwelling pulses within seasons, as well as the effects of El Niño events on marine habitat use associated with variability in prey distribution (Soto et al., 2006). Climate predictions could additionally be integrated in the bycatch risk framework to predict future interaction scenarios under changing ocean conditions (García‐Reyes et al., 2023).

### 4.6 Conclusions

This research is the first to quantify habitat associations for SASL in northern Chile and to characterize the risk of bycatch in significant regional fisheries. The population‐level and individual‐level habitat models demonstrated broad potential suitability in cool, nearshore environments, while individual‐level occurrence was restricted by distance‐based movement potential. The combined distribution map indicates suitable habitat for females and juveniles, highlighting areas near the tagging site of Iquique that are likely to be occupied at the individual level. The areas of greater population‐level distribution reveal opportunities for additional local research to refine the prediction of potential suitability. Our bycatch risk assessment identified low risk for both industrial and artisanal fleets. However, the cumulative risk map highlights the 5 NM artisanal fishing zone boundary as an area of overlap for both fleets, suggesting this boundary is a critical area upon which to focus interaction mitigation efforts. This research provides a new framework for assessing interaction risk to mobile species by integrating telemetry‐based suitability models with a bycatch risk assessment.

## AUTHOR CONTRIBUTIONS

**Bethany H. Frantz:** Conceptualization (lead); data curation (equal); formal analysis (lead); investigation (lead); methodology (lead); project administration (lead); visualization (lead); writing – original draft (lead); writing – review and editing (lead). **Maritza Sepúlveda:** Conceptualization (equal); funding acquisition (lead); investigation (equal); resources (lead); writing – review and editing (equal). **Marisol García‐Reyes:** Data curation (equal); investigation (supporting); writing – review and editing (supporting). **Rodrigo Vega:** Data curation (equal); investigation (equal); writing – review and editing (supporting). **Daniel M. Palacios:** Writing – review and editing (equal). **Luis Bedriñana‐Romano:** Writing – review and editing (equal). **Luis A. Hückstädt:** Investigation (equal). **Macarena Santos‐Carvallo:** Investigation (supporting); resources (equal). **Jerry D. Davis:** Conceptualization (supporting); methodology (supporting); writing – review and editing (equal). **Ellen Hines:** Conceptualization (equal); funding acquisition (lead); writing – review and editing (equal).

## FUNDING INFORMATION

This research was funded by the Lenfest Ocean Program (Contract 00034562). L.B. was supported by COPAS Coastal ANID FB210021.

## CONFLICT OF INTEREST STATEMENT

The authors declare that they have no competing interests.

## Supporting information


**Tables S1** **–S4**
Figures S1–S16

## Data Availability

Sources of environmental data are outlined in the methods section. Telemetry data are available here https://doi.org/10.5281/zenodo.7889063. Due to the restricted and sensitive nature of the fishing activity data, requests for these datasets should be directed to Bethany Frantz (bntyhf@gmail.com).
