# Supplementary material for: Combining potential and realized distribution modeling of telemetry data for a bycatch risk assessment
Source: Ecol Evol. 2024 Jun 25;14(6):e11541. doi: 10.1002/ece3.11541 (PMC11199131; doi:10.1002/ece3.11541)
Supplement: Supplementary file 1 — Tables S1 –S4 Figures S1–S16 [file ECE3-14-e11541-s001.docx]

**Supplementary Information for:**

**Combining potential and realized distribution modeling of telemetry data for a bycatch risk assessment**

**Contents**

[1. Trip Characteristics 1](#_Toc166404228)

[2. Habitat Models 1](#_Toc166404229)

[2.1. Variable Selection 1](#_Toc166404230)

[2.2. Pseudo-Absence Generation 3](#_Toc166404231)

[2.3. GLM Spatial Cross-Validation Methods 4](#_Toc166404232)

[2.4. Data Reduction Methods 5](#_Toc166404233)

[2.5. Model Evaluation 6](#_Toc166404234)

[2.5.1. Full and Reduced Model Performance 6](#_Toc166404235)

[2.5.2. Residual Autocorrelation 7](#_Toc166404236)

[2.5.2.1. Population Model (GLM) 8](#_Toc166404237)

[2.5.2.2. Individual Models (GLMM) 9](#_Toc166404238)

[2.5.3. Population Model (GLM) Variable Importance 10](#_Toc166404239)

[3. Bycatch Risk Assessment 10](#_Toc166404240)

[3.1. Criteria Scores 10](#_Toc166404241)

[3.2. Spatially Explicit Criteria 13](#_Toc166404242)

[3.2.1. Fishing Intensity 13](#_Toc166404243)

[3.2.2. Habitat suitability 14](#_Toc166404244)

[3.2.3. Likelihood of interaction 14](#_Toc166404245)

[3.3. Data Uncertainty 16](#_Toc166404246)

[**References** 18](#_Toc166404247)

## Trip Characteristics

To analyze trips made by tracked sea lions, a single trip was defined as the sequence of at-sea locations between haul out events, starting and ending on land (Jones et al. 2015, Villegas-Amtmann et al. 2008). The SRDL tags recorded the start and end times of haul-out behavior using wet/dry sensors. We combined these haul-out event data with location data by matching individual IDs and timestamps. All haul-outs were assigned to a coordinate location. Unique trips were identified using the haul-out records and visually inspecting mapped tracks to identify arrival and departure from colonies. An origin and destination haul-out were designated for all trips (Jones et al. 2015). Using the R package ‘*trip*’ (Sumner 2011), we calculated mean trip duration and mean distance per trip. Only trips with greater than two at-sea locations were used for trip statistics to avoid including instances in which an animal entered the water but did not make an actual trip.

In total, we identified 215 unique at-sea trips, with an average travel distance of 90 km (± 71 km; Table S1). The locations were on average 14.6 km (± 14.1 km) from shore, with a maximum distance of 129 km from shore by an adult female. All animals remained north of Mejillones Peninsula and most locations were concentrated near the tagging site at Punta Patache.

**Table S1.** Description, tracking information, and trip characteristics (mean ± SD) of nine tagged South American sea lions from Punta Patache, Chile.

| ID | Sex | Age class | Days tracked | Mean trip duration (days) | Mean trip distance (km) |
| --- | --- | --- | --- | --- | --- |
| 96721 | M | Juvenile | 71 | 1.69 ± 1.08 | 48.87 ± 46.26 |
| 96722 | F | Juvenile | 41 | 1.36 ± 1.0 | 52.25 ± 45.23 |
| 96723 | F | Adult | 102 | 1.69 ± 0.72 | 59.01 ± 31.15 |
| 96724 | F | Adult | 32 | 1.60 ± 1.05 | 86.74 ± 47.60 |
| 96726 | M | Juvenile | 31 | 2.12 ± 1.27 | 147.17 ± 107.87 |
| 96727 | F | Adult | 77 | 1.79 ± 0.91 | 80.26 ± 56.04 |
| 96728 | F | Adult | 104 | 2.70 ± 1.02 | 122.99 ± 52.37 |
| 96729 | F | Adult | 51 | 1.96 ± 1.05 | 88.58 ± 47.36 |
| 96730 | F | Adult | 125 | 2.51 ± 1.44 | 154.70 ± 96.64 |

## Habitat Models

### Variable Selection

We chose eight candidate variables for habitat modeling (Table S2). Prior to model development we tested the covariates for collinearity using Pearson’s correlation coefficient and additionally calculated variance inflation factors during model fitting. Distance to shelf and distance to shore had high pairwise Pearson’s correlation coefficient values with Chl and bathymetry (Figure S1), so we removed both from the candidate variables. In the GLM development, Chl and SST had a high VIF (>4.5). We built separate models using each of these variables and chose the model with the higher AUC and CBI across cross-validation folds. The final GLM included Chl, distance to rivers, bathymetry, slope, and EKE. The final GLMM included SST, slope, and the additional dispersal kernel (DK) variable.

**Table S2.** List and description of all environmental predictor variables with native resolution and data sources.

| Variable | Description | Spatial Resolution | Temporal Resolution | Source |
| --- | --- | --- | --- | --- |
| **Seafloor depth (m)** | Bathymetric depth | 15 arc-second |  | GEBCO Compilation Group 2021 ([www.gebco.net](https://www.gebco.net)) |
| **Slope (°)** | Steepness derived from bathymetry |  |  | GIS derived |
| **Distance to shelf** | Euclidean distance to continental shelf |  |  | GIS derived  (Shelf feature retrieved from [www.bluehabitats.org](https://www.bluehabitats.org/); Harris et al. 2014) |
| **Distance to coast** | Euclidean distance to shoreline |  |  | GIS derived |
| **Distance to rivers** | Euclidean distance to river-mouths |  |  | GIS derived (River features retrieved from [data.humdata.org/dataset/hotosm_chl_waterways](https://data.humdata.org/dataset/hotosm_chl_waterways); HOT-OSM 2020) |
| **Sea surface temperature (°C)** | Mean temperature | 1 km^2^ | Obtained at 1-month | Chin et al. 2017  (<https://registry.opendata.aws/mur/>) |
| **Chlorophyll-α (mg/m^3^)** | Proxy for marine productivity | 4 km^2^ | Obtained at 1-month | Sathyendranath et al. 2020 (<https://catalogue.ceda.ac.uk/uuid/99348189bd33459cbd597a58c30d8d10>) |
| **Eddy kinetic energy (cm^2^/s^2^**) | Measure current activity, derived from sea surface height anomalies (SSHA) | 1/6th degree | SSHA obtained at 5-day | Derived (SSHA data retrieved from Zlotnicki et al. 2019) |


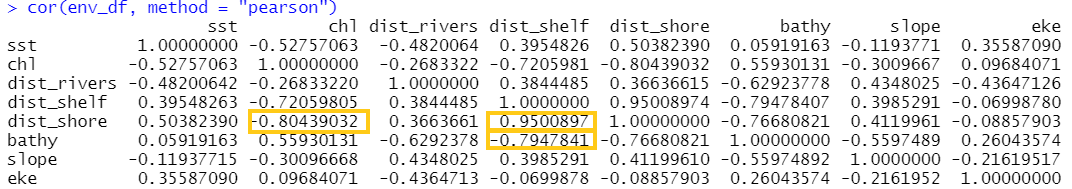


**Figure S1.** Pearson’s correlation coefficient matrix for the candidate environmental variables with highly correlated variables highlighted by yellow boxes.

### Pseudo-Absence Generation

We used an environmental dissimilarity approach for pseudo-absence generation to restrict background locations to areas less likely to feature false absences. For the population-level approach, pseudo-absences were created further from presence points (>5 km) and in more environmentally dissimilar locations (Figure S2) than for the individual approach (>2 km; Figure S3), based on a presence-only profiling algorithm (one-class support vector machine) of the study area.


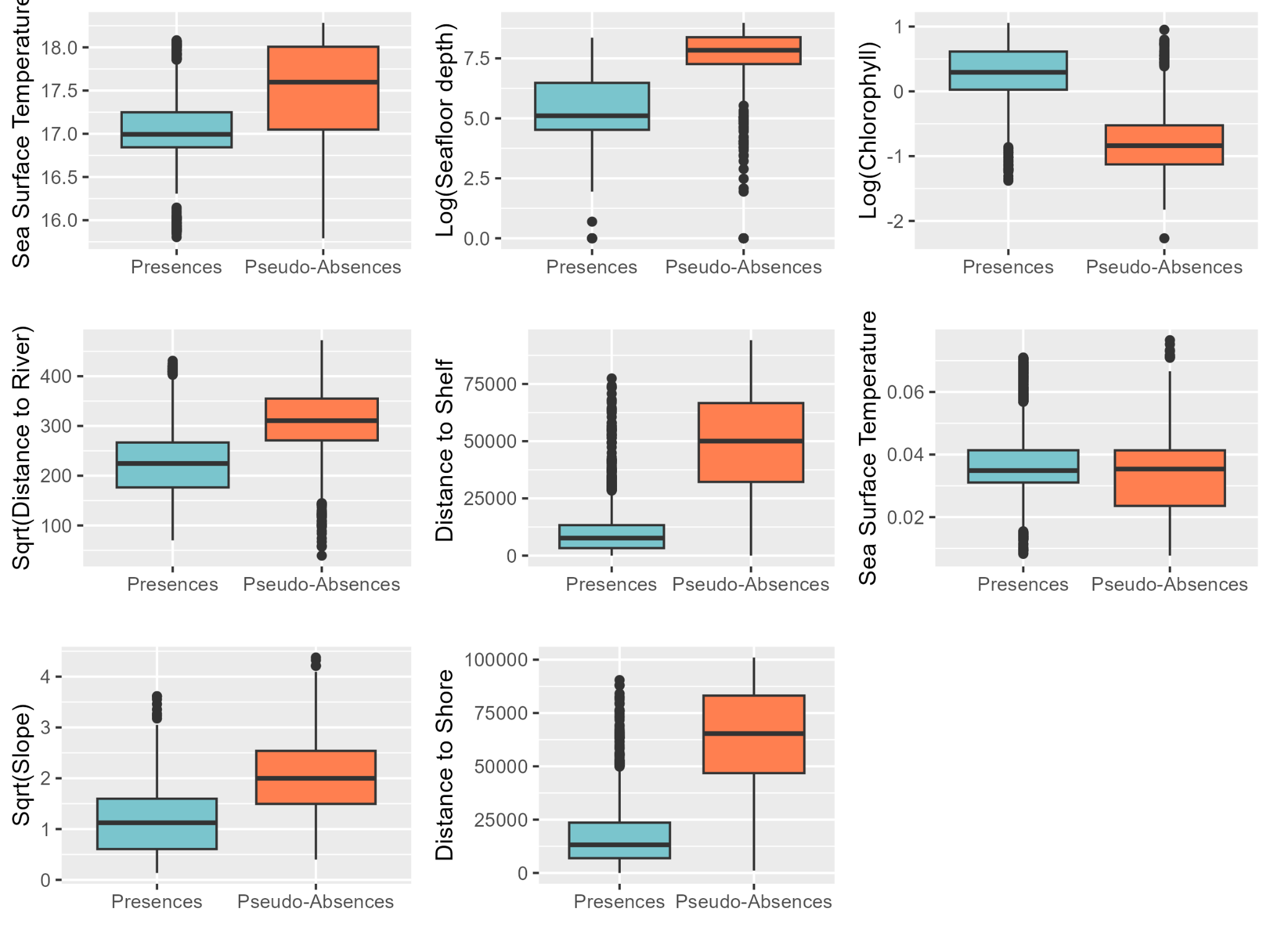


Figure S2. The distribution of environmental variables at presence and pseudo-absence locations for the population-level approach.


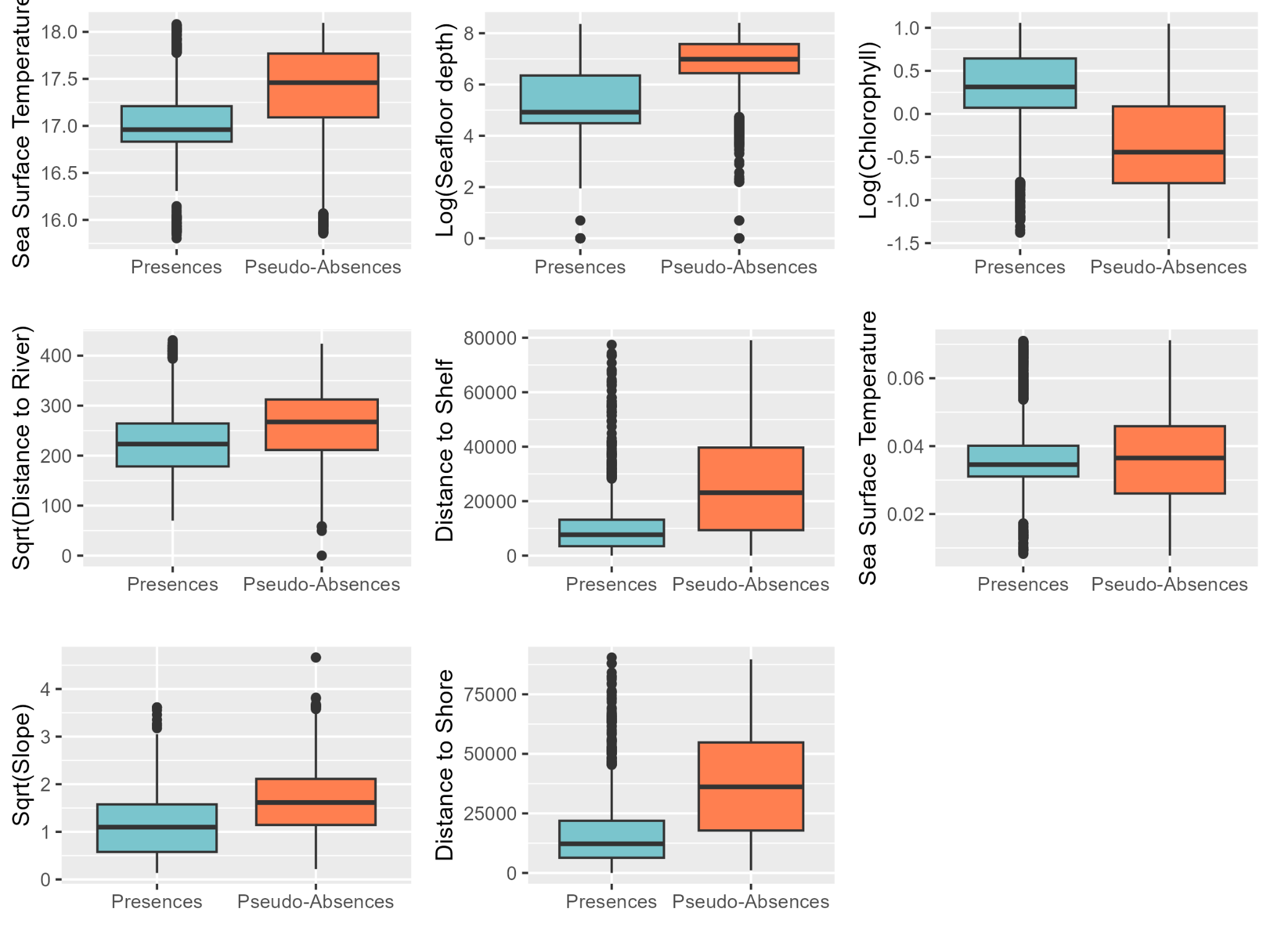


Figure S3. The distribution of environmental variables at presence and pseudo-absence locations for the individual-level approach.

### GLM Spatial Cross-Validation Methods

Because tracking data exhibit an internal dependence structure, for the population model (GLM), we used a spatial blocking cross-validation procedure (Robinson et al. 2021). Using the R package ‘*blockCV*’ (Valavi et al. 2019), we estimated the optimal block size based on the range of autocorrelation in the environmental variables (Figure S4).


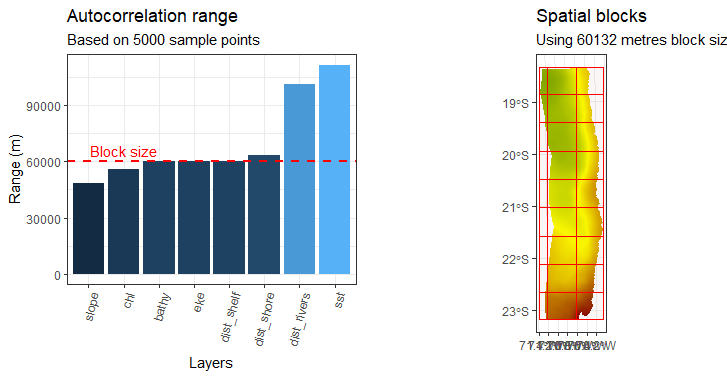


**Figure S4.** The range of autocorrelation of environmental variables based on 5000 randomly sampled points (left) and the spatial blocks used for model cross-validation overlaid on the SST raster (right), from the R package ‘*blockCV*’.

### Data Reduction Methods

The final habitat models were developed with spatially rarefied occurrences (‘full’ data model). However, we additionally tested a reduced occurrence dataset (‘reduced’ data model) in an effort to eliminate bias from sampling intensity (i.e., variation in the number of locations per individual) and to further reduce serial autocorrelation. In the reduced data model, for each individual we randomly selected ten tracking days and retained three locations per day, yielding 30 locations per individual (Briscoe et al. 2018, Heide‐Jørgensen et al. 2020). This approach was chosen to maximize the number of occurrences per individual, without eliminating animals with low individual sample sizes. Performance metrics of models using this reduced dataset were compared to those of the full (grid-sampled) dataset.

### Model Evaluation

#### Full and Reduced Model Performance

The down-sampling scheme reduced the dataset by 90%. Model performance metrics between full (grid-sampled) and reduced datasets differed more in the individual model than the population model (Figure S5, S6). For the population model (GLM), both the full and reduced data models had a relatively high CBI: median 0.95 and 0.79, respectively, indicating suitability predictions are consistent with presences in the test data. AUC was similar between data models, median 0.96 for both, but the reduced model had a slightly larger standard deviation (Figure S5). In the individual models (GLMM), CBI was higher in the full data model than the reduced (median 0.89 and 0.62, respectively) but AUC did not differ strongly (Figure S6).


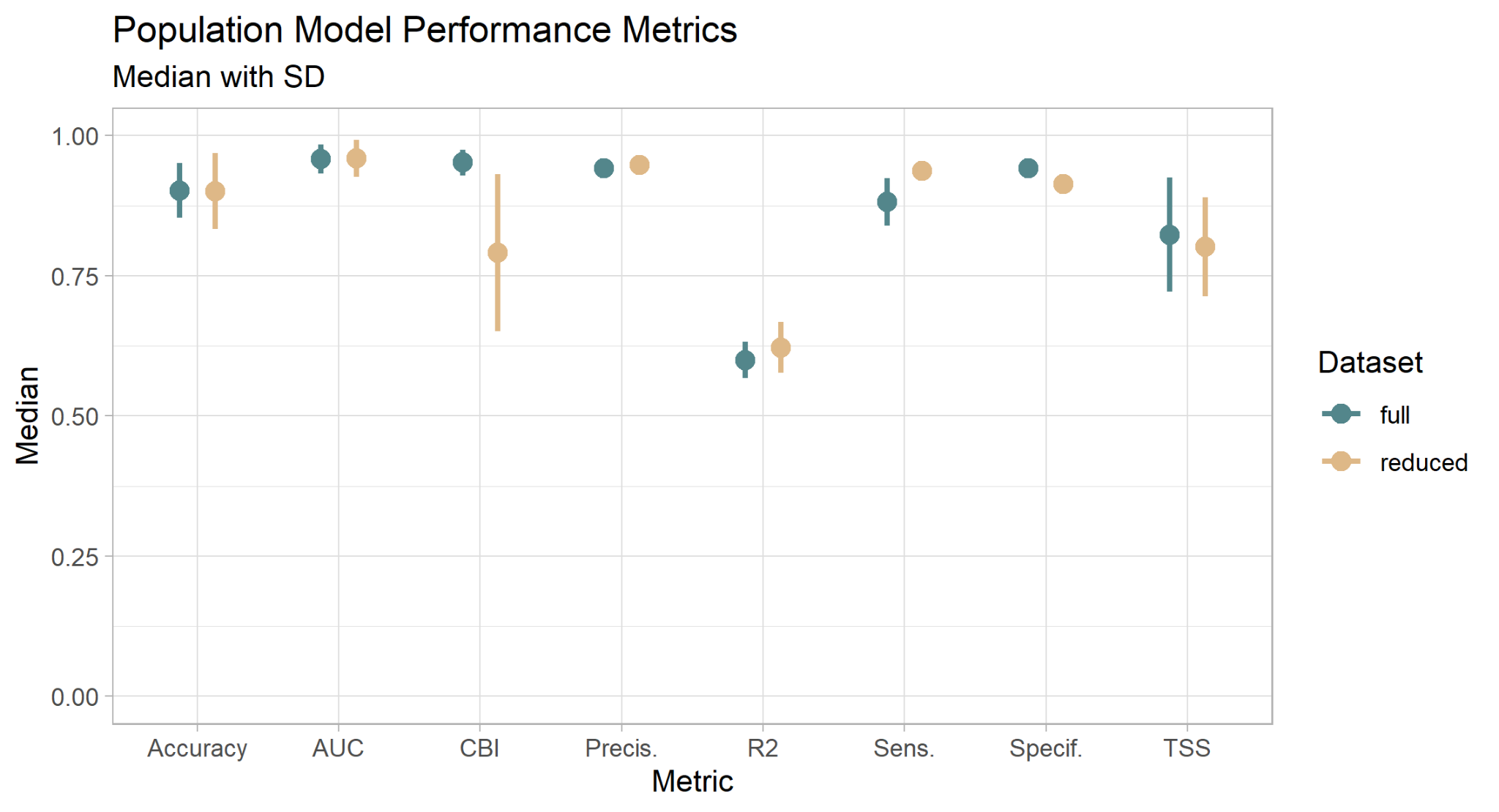


**Figure S5.** Performance metrics for the population model (median and standard deviation from five spatial cross-validation runs) using the full (grid-sampled) and reduced datasets.


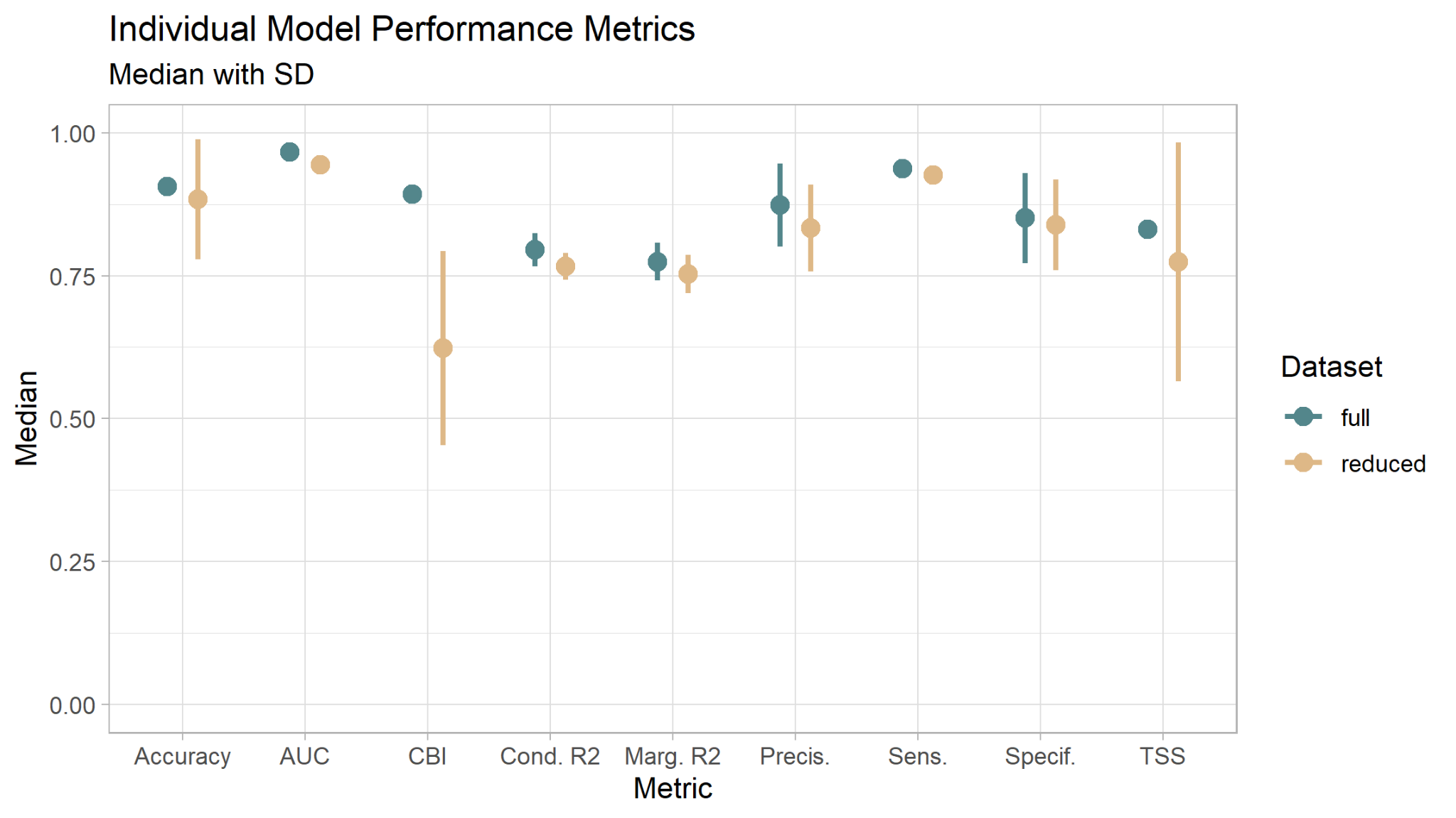


**Figure S6.** Performance metrics for the individual model (median and standard deviation across nine individual exclusion cross-validation runs) using the full (grid-sampled) and reduced datasets.

#### Residual Autocorrelation

We created variograms of model residuals to examine autocorrelation of the full and reduced data models. For the population model, variogram range indicates that autocorrelation does not extend beyond 36km for the full dataset, and 35km for the reduced (Figure S7, Figure S8, Figure S9). In the individual model, variogram range was higher with the reduced dataset (26 km) than full dataset (14 km), but with a lower sill (Figure S10, Figure S11, Figure S12).

Because our goal was to predict suitability within a set study area, we prioritized the representativeness of locations in our models (Fieberg 2007). Overall, the full data models performed marginally better than the reduced and data reduction did not substantially reduce autocorrelation. We, therefore, chose the full data models as the final estimate of suitability.

##### Population Model (GLM)


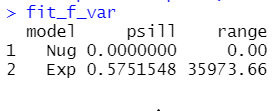

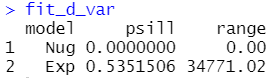


**Figure S7.** Variogram fits, indicating the range of autocorrelation (m) for the population model with full (left) and reduced (right) datasets.


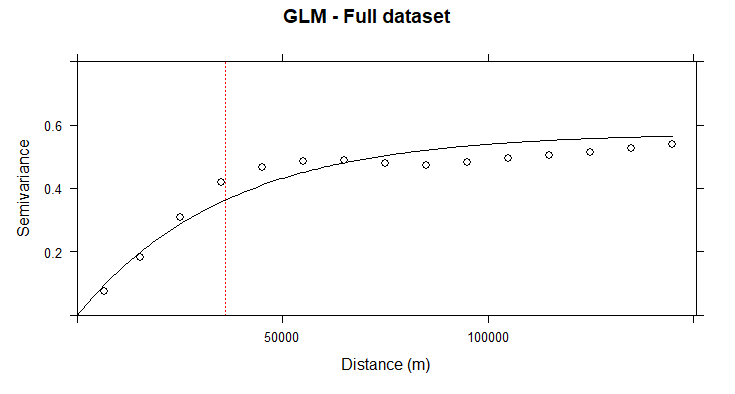


**Figure S8.** Semivariogram of model residuals from the GLM of the full dataset. Distance between location pairs (x-axis) are plotted against semivariance (y-axis).


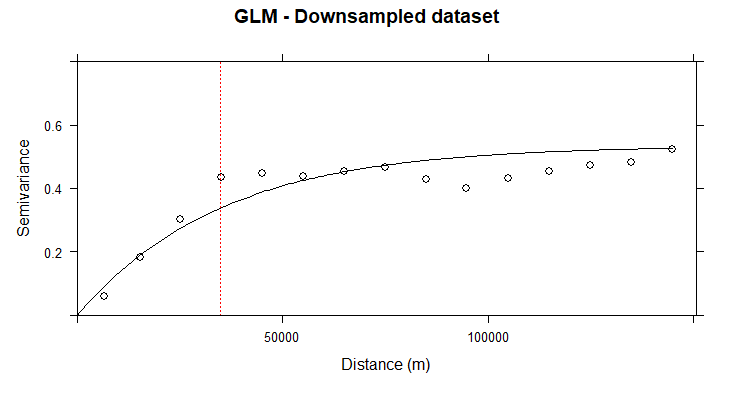


**Figure S9.** Semivariogram of model residuals from the GLM of the downsampled dataset. Distance between location pairs (x-axis) are plotted against semivariance (y-axis).

##### Individual Models (GLMM)


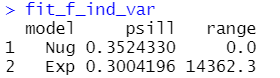

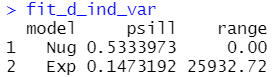


**Figure S10.** Variogram fits, indicating the range of autocorrelation (m) for the individual model with full (left) and reduced (right) datasets.


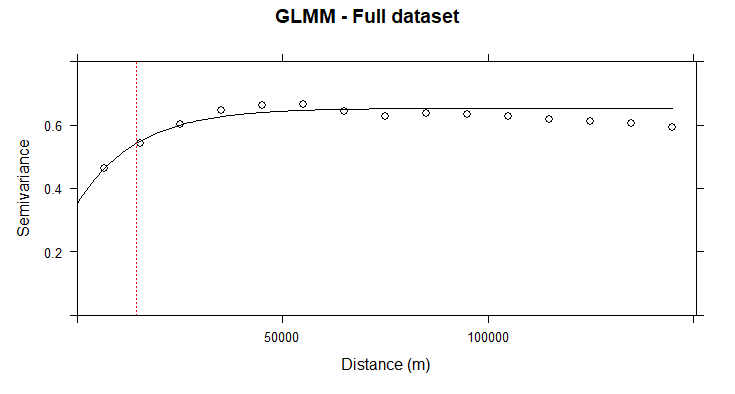


**Figure S11.** Semivariogram of model residuals from the GLMM of the full dataset. Distance between location pairs (x-axis) are plotted against semivariance (y-axis).


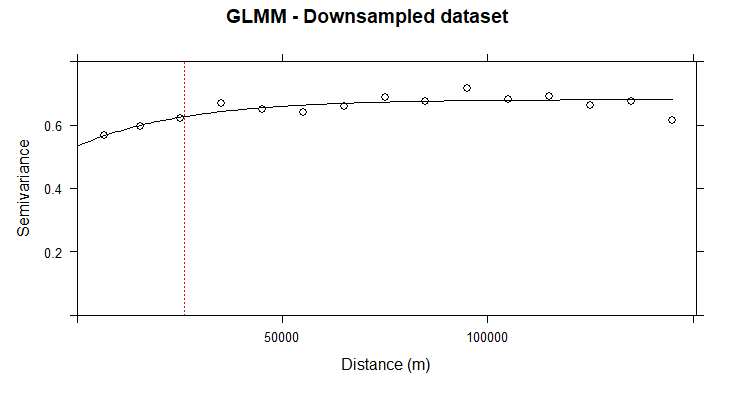


**Figure S12.** Semivariogram of model residuals from the GLMM of the downsampled dataset. Distance between location pairs (x-axis) are plotted against semivariance (y-axis).

#### Population Model (GLM) Variable Importance

We used the absolute value of the t-statistic for each variable to determine relative importance within the linear model (Figure S13).

**
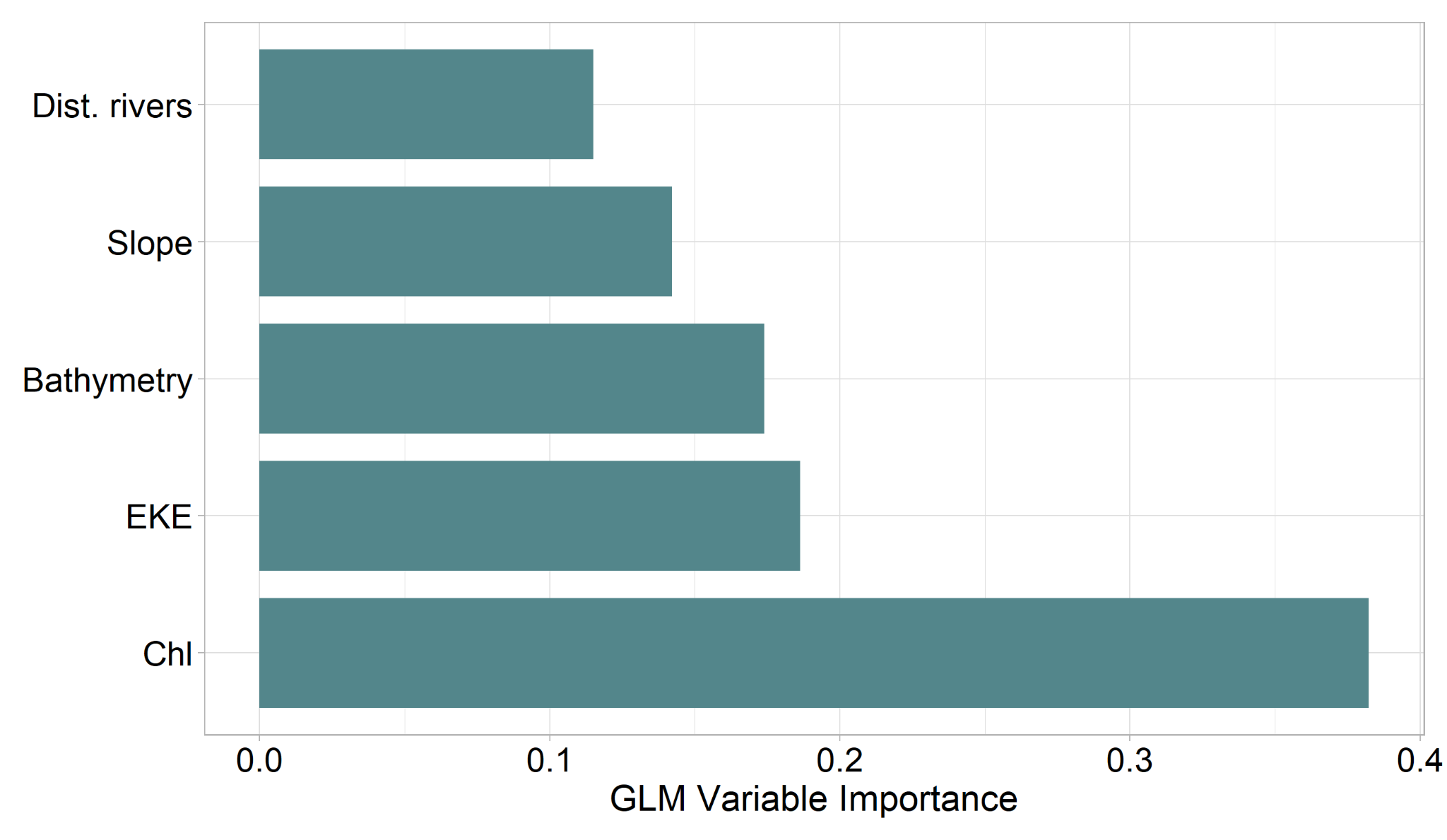
**

**Figure S13.** Importance (relative contribution) of environmental variables in the final population model.

## Bycatch Risk Assessment

### Criteria Scores

The criteria ratings, data quality, and weight scores for the Bycatch Risk Assessment (ByRA) model were decided through a series of online, collaborative workshops. All co-authors, who have specialized knowledge on the fisheries and species of concern, agreed upon ratings based on existing data and research and professional expertise (Table S2).

**Table S2.** Bycatch risk assessment criteria scores

Tables of exposure (top) and consequence (bottom) criteria scores used in the Bycatch Risk Assessment model, based on the definitions and guidelines presented in Verutes et al. (2020). Ratings indicate level of risk associated with a criterion: high (3), medium (2), low (1), or spatially explicit criteria (SEC). The data quality (DQ) scores range from limited/poor quality (3) to best data available (1). Weight scores the significance of a criterion from least important (1) to most (3). Scores were decided through discussions with all co-authors and fishery experts from the Instituto Fomento Pesquero, Chile.

| **Exposure Criteria** | | | | | | | | | | | | | |  |  |  |  |
| --- | --- | --- | --- | --- | --- | --- | --- | --- | --- | --- | --- | --- | --- | --- | --- | --- | --- |
|  | | **Industrial Purse-Seine** | | | | | | **Artisanal Purse-Seine** | | | | | |  |  |  |  |
| **Criterion** | | | **Rating** | **DQ** | | | **Weight** | **Rating** | **DQ** | | | **Weight** | |  |  |  |  |
| Likelihood of capture by gear | | | 3 | 2 | | | 2 | 2 | 2 | | | 2 | |  |  |  |  |
| Temporal overlap | | | 2 | 2 | | | 2 | 2 | 2 | | | 2 | |  |  |  |  |
| Current status of management | | | 2 | 2 | | | 2 | 2 | 2 | | | 2 | |  |  |  |  |
| Intensity of gear use | | | SEC | 2 | | | 2 | SEC | 3 | | | 2 | |  |  |  |  |
| Likelihood of interaction with gear | | | SEC | 2 | | | 2 | SEC | 3 | | | 2 | |  |  |  |  |
| **Consequence Criteria** | | | | | | | | | | | | | | | | | |
| **Resilience** | | | | | | | | **Sensitivity** | | | | | | | | | |
|  |  |  |  |  |  |  |  |  | | **Industrial Purse-Seine** | | | | | **Artisanal Purse-Seine** | | |
| **Criterion** | **Rating** | | | | **DQ** | **Weight** | | **Criterion** | | | **Rating** | | **DQ** | **Weight** | **Rating** | **DQ** | **Weight** |
| Age of maturity | | 2 | | | 2 | 2 | | Mortality from gear | | | 1 | | 2 | 2 | 1 | 2 | 2 |
| Reproductive strategy | | 2 | | | 2 | 2 | | Life stages affected by gear | | | 2 | | 3 | 2 | 1 | 3 | 2 |
| Population connectivity | | 2 | | | 2 | 2 | |  | | |  | |  |  |  |  |  |
| Local species status | | 1 | | | 2 | 2 | |  | | |  | |  |  |  |  |  |

#### Spatially Explicit Criteria

##### Fishing Intensity

To estimate fishing intensity, we used 2010-2015 fishing point data, corresponding to the time period of the animal tracking data. The data were shared through the Institute of Fisheries Development’s monitoring program for the main pelagic fisheries in the northern zone of Chile. Industrial fishing set data were recorded by scientific observers. The artisanal data have greater uncertainty in coverage and geography, as these points were assigned to the center-points of a 1 x 1 nautical mile grid, based on reports of landings from the artisanal fleet. We used a kernel density estimation with a 3 km cell size to accommodate uncertainty, and reclassified values using quantiles (Figure S14).


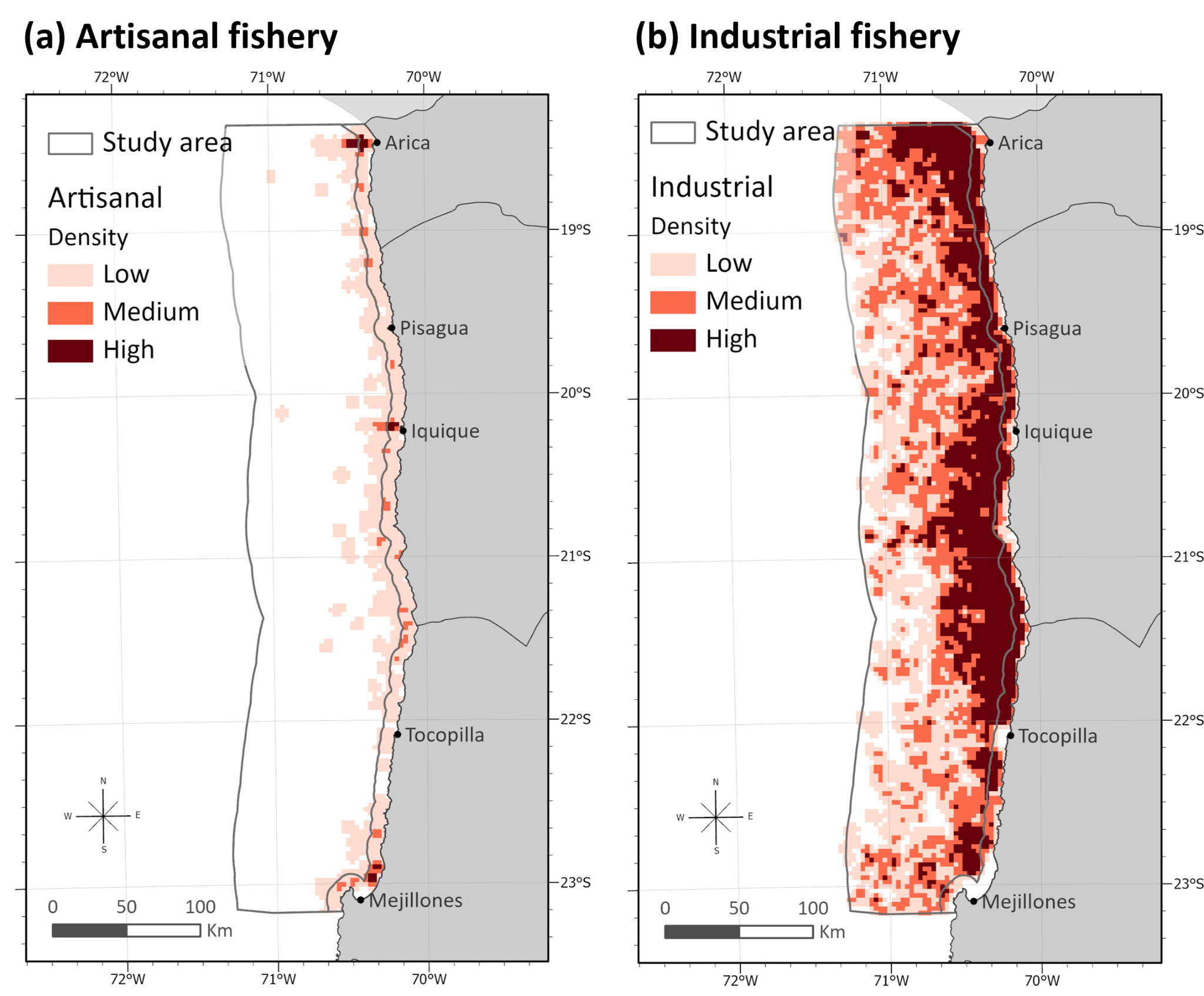


**Figure S14.** Maps of the fishing intensity layers (kernel density of fishing sets per 3 km) for the northern artisanal (a) and industrial (b) purse-seine fisheries, classified low to high.

##### Habitat suitability

In ByRA, a habitat suitability or species distribution layer, is used to estimate the likelihood of interaction between the species and stressors, across the study area. For this purpose, we reclassified the combined suitability map from the population and individual models. First, we used quantiles to classify the potential and realized distribution predictions. We then classified each cell in the combined map as high, medium, or low overall suitability (Figure S15a). The reclassification schema was based on the suggestions in Chambault et al. (2021) and modified for our system to emphasize areas with high potential distribution (Figure S15).


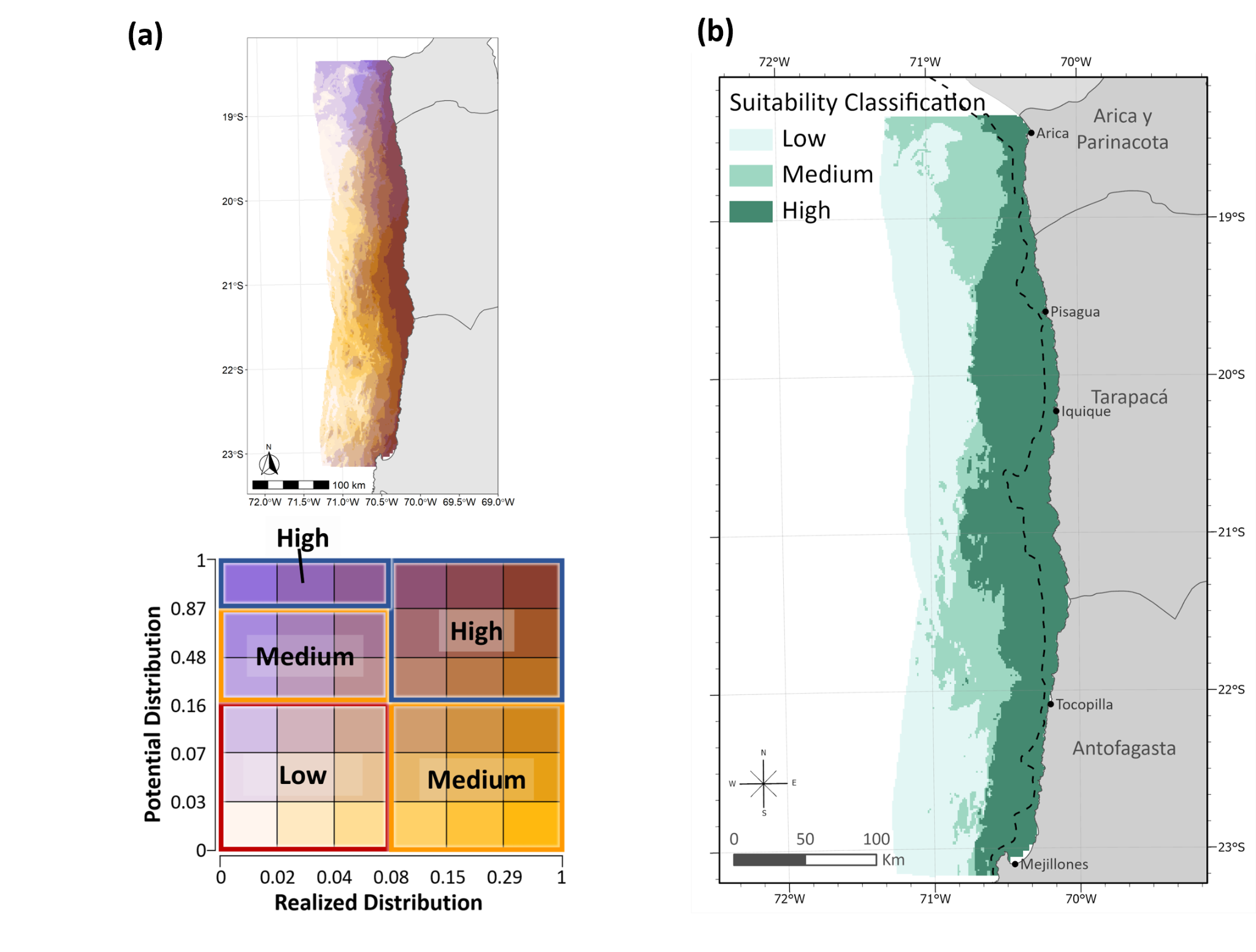


**Figure S15.** (a) The combined distribution map and suitability reclassification schema and (b) the reclassified combined suitability map. The blue boxes in the classification schema indicate classification of high combined suitability, yellow represents medium suitability, and red indicates low.

##### Likelihood of interaction

To estimate likelihood of interaction between fishing activity and SASL, we summed the combined SASL habitat suitability layer (Figure S15b) and fishery intensity rasters (Figure S14; Hines et al. 2020). The summed values were reclassified as follows: low (2-3), medium (4), and high (5-6) (Figure S16).


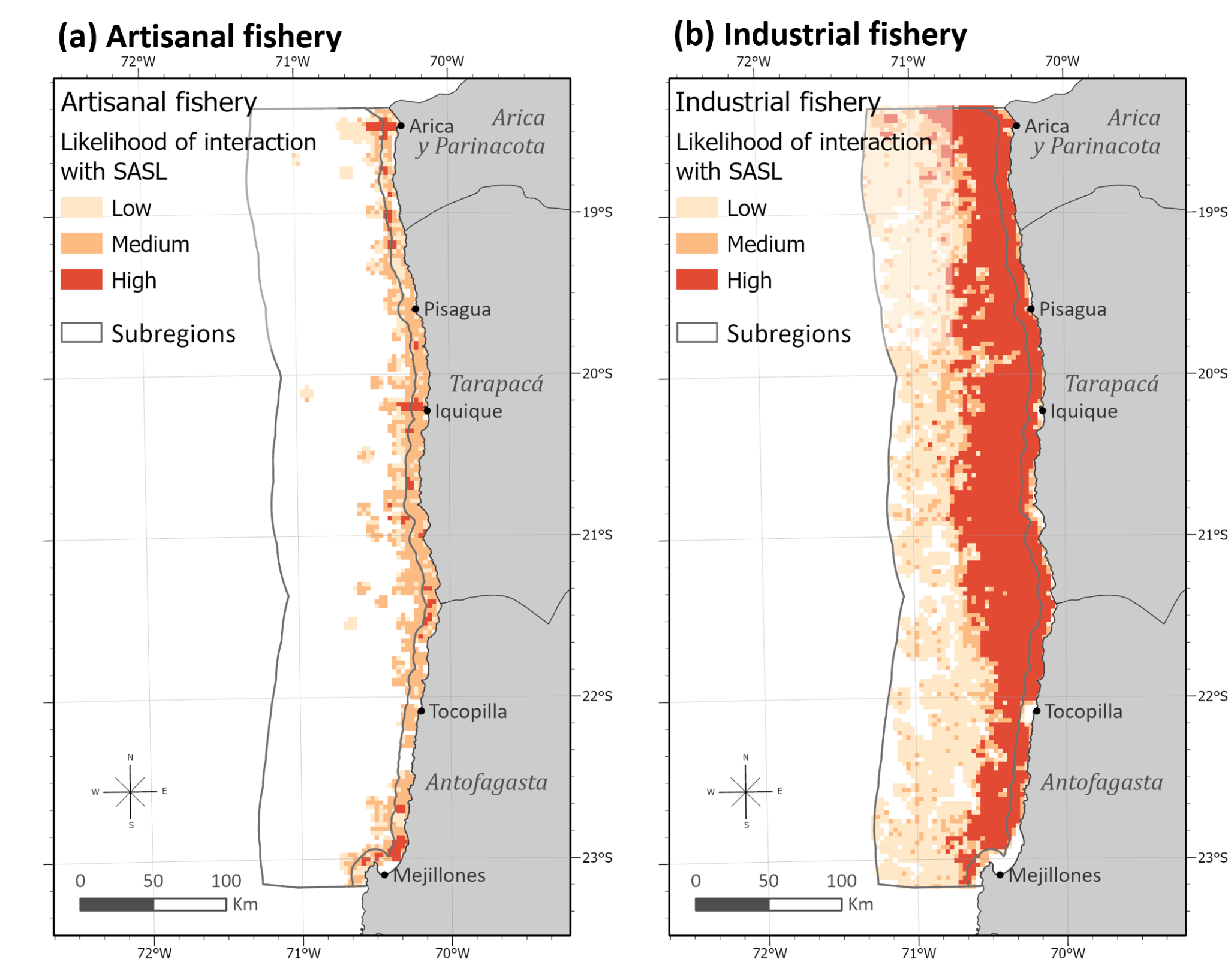


**Figure S16.** Likelihood of interaction maps of female and juvenile South American sea lions with artisanal (a) and industrial (b) purse-seine fisheries.

### Data Uncertainty

In consideration of the variety of data sources in the bycatch model, the ByRA framework uses a stoplight approach to characterize data uncertainty. We used the criteria outlined in Hines et al. (2020) and Verutes et al. (2020) to assess the data in our risk model (Table S3). Both the animal data and habitat suitability were classified as having low uncertainty because of the quality of the telemetry data and performance of the habitat models (Table S4). Fishing effort data were a combination of low uncertainty data from scientific observers (industrial fleet) and higher uncertainty fishing logs (artisanal fleet). Finally, bycatch data has medium uncertainty as estimates were not available for the period of this study but previous research on the northern purse-seine fleets have been published (González et al. 2015).

**Table S3**. Criteria for categorizing data uncertainty in the ByRA model, based on Hines et al. (2020)

| **Information Type** | **Green** | **Yellow** | **Red** |
| --- | --- | --- | --- |
| **Animal Data** | Data collected in formal surveys | Sightings/photo ID collected during opportunistic surveys | Few sightings collected, or only available from interviews; no abundance estimation possible |
| **Habitat Suitability** | Estimated from modeling with environmental data | Rule-based estimation, minimal environmental variables collected | Criteria from other regions used to estimate animal distribution |
| **Fishing effort data** | Surveyed fishing occurrence per unit of effort, distance, or time | Spatial and/or temporal distribution of fishing based on interviews or expert opinion | Sparse or incomplete data, no geospatial or precise localization of fishing effort/gear |
| **Bycatch data** | Data available from onboard observers, interviews, or stranding data; estimation of bycatch rate possible | Relative estimation of bycatch from interviews or stranding data | No estimate of bycatch or strandings available |

**Table S4.** Classification of data uncertainty in the SASL and purse-seine fishery ByRA model, using the stoplight approach and classification schema from Table S3.

| **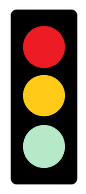** | **Criteria** | **Description** |
| --- | --- | --- |
|  | **Animal Data** | Satellite telemetry |
|  | **Habitat Suitability** | Generalized linear and mixed-effects models |
|  | **Fishing effort data** | Scientific observes (~5% coverage) and generalized artisanal fleet landing reports |
|  | **Bycatch data** | Interaction rate estimate from González et al. (2015) |

## **References**

Briscoe, D. K., Fossette, S., Scales, K. L., Hazen, E. L., Bograd, S. J., Maxwell, S. M., McHuron, E. A., Robinson, P. W., Kuhn, C., Costa, D. P., Crowder, L. B., & Lewison, R. L. (2018). Characterizing habitat suitability for a central-place forager in a dynamic marine environment. *Ecology and Evolution*, *8*(5), 2788–2801. <https://doi.org/10.1002/ece3.3827>

Chambault, P., Hattab, T., Mouquet, P., Bajjouk, T., Jean, C., Ballorain, K., Ciccione, S., Dalleau, M., & Bourjea, J. (2021). A methodological framework to predict the individual and population-level distributions from tracking data. *Ecography, 44*(5), 766–777. <https://doi.org/10.1111/ecog.05436>

Chin, T. M., Vazquez-Cuervo, J., & Armstrong, E. M. (2017). A multi-scale high-resolution analysis of global sea surface temperature. *Remote sensing of environment*, *200*, 154-169.

Fieberg, J. (2007). Kernel density estimators of home range: Smoothing and the autocorrelation red herring. *Ecology, 88*(4), 1059–1066. <https://doi.org/10.1890/06-0930>

GEBCO Compilation Group (2021) GEBCO 2021 Grid. doi:10.5285/c6612cbe-50b3-0cff-e053-6c86abc09f8f

González, A., Vega, R., & Yáñez, E. (2015). Operational interactions between the South American sea lion Otaria flavescens and purse seine fishing activities in northern Chile. *Revista de Biologia Marina y Oceanografia, 50*(3), 479–489. <https://doi.org/10.4067/S0718-19572015000400007>

Harris, P.T., Macmillan-Lawler, M., Rupp, J. and Baker, E.K. 2014. Geomorphology of the oceans. *Marine Geology*, 352: 4-24.

Heide-Jørgensen, M. P., Blackwell, S. B., Williams, T. M., Sinding, M. H. S., Skovrind, M., Tervo, O. M., Garde, E., Hansen, R. G., Nielsen, N. H., Ngô, M. C., & Ditlevsen, S. (2020). Some like it cold: Temperature-dependent habitat selection by narwhals. *Ecology and Evolution*, *10*(15), 8073–8090. https://doi.org/10.1002/ece3.6464

 Hines, E., Ponnampalam, L. S., Junchompoo, C., Peter, C., Vu, L., Huynh, T., Caillat, M., Johnson, A. F., Minton, G., Lewison, R. L., Verutes, G. M., & Kiszka, J. (2020). Getting to the bottom of bycatch: a GIS-based toolbox to assess the risk of marine mammal bycatch. *Endangered Species Research, 42*, 37–57. <https://doi.org/10.3354/esr01037>

HOT-OSM (Humanitarian OpenStreetMap Team). (2020). Chile Waterways (Updated July 2020) [Spatial data file]. Retrieved from <https://data.humdata.org/dataset/hotosm_chl_waterways>.

Jones EL, McConnell BJ, Smout S, Hammond PS, Duck CD, Morris CD, Thompson D, Russel DJF, Vincent C, Cronin M, Sharples RJ, Matthiopoulos J (2015) Patterns of space use in sympatric marine colonial predators reveal scales of spatial partitioning. Mar Ecol Prog Ser 534:235–249.

Robinson, C. L., Proudfoot, B., Rooper, C. N., & Bertram, D. F. (2021). Comparison of spatial distribution models to predict subtidal burying habitat of the forage fish Ammodytes personatus in the Strait of Georgia, British Columbia, Canada. *Aquatic Conservation: Marine and Freshwater Ecosystems*, *31*(10), 2855-2869.

Sathyendranath, S., Jackson, T., Brockmann, C., Brotas, V., Calton, B., Chuprin, A., Clements, O., Cipollini, P., Danne, O., Dingle, J., Donlon, C., Grant, M., Groom, S., Krasemann, H., Lavender, S., Mazeran, C., Mélin, F., Moore, T.S., Müller, D., Regner, P., … & Platt, T. (2020). ESA Ocean Colour Climate Change Initiative (Ocean_Colour_cci): Global chlorophyll-a data products gridded on a sinusoidal projection, Version 4.2. Centre for Environmental Data Analysis. [Data file] Retrieved March 10, 2022 from <https://catalogue.ceda.ac.uk/uuid/99348189bd33459cbd597a58c30d8d10>

Sumner, M. D. (2011). The Tag Location Problem. [Doctoral dissertation]. University of Tasmania. <https://eprints.utas.edu.au/12273/3/sumner.pdf>

Valavi, R., Elith, J., Lahoz-Monfort, J. J., & Guillera-Arroita, G. (2019). blockCV: An r package for generating spatially or environmentally separated folds for k-fold cross-validation of species distribution models. *Methods in Ecology and Evolution*, *10*(2), 225–232. https://doi.org/10.1111/2041-210X.13107

Verutes, G. M., Johnson, A. F., Caillat, M., Ponnampalam, L. S., Peter, C., Vu, L., Junchompoo, C., Lewison, R. L., & Hines, E. M. (2020). Using GIS and stakeholder involvement to innovate marine mammal bycatch risk assessment in data-limited fisheries. *PLoS ONE, 15*(8 August), 1–25. <https://doi.org/10.1371/journal.pone.0237835>

Villegas-Amtmann, S., Costa, D. P., Tremblay, Y., Salazar, S., & Aurioles-Gamboa, D. (2008). Multiple foraging strategies in a marine apex predator, the Galapagos sea lion Zalophus wollebaeki. *Marine Ecology Progress Series*, *363*, 299-309.

Zlotnicki, V., Qu, Z., Willis, J., (2019). MEaSUREs Gridded Sea Surface Height Anomalies Version 1812. PO.DAAC, CA, USA. [Data file]. Retrieved May 1, 2022 from <https://doi.org/10.5067/SLREF-CDRV2>
